# Axon-Schwann cell interactions during peripheral nerve regeneration in zebrafish larvae

**DOI:** 10.1186/1749-8104-9-22

**Published:** 2014-10-17

**Authors:** Maria Laura Ceci, Camila Mardones-Krsulovic, Mario Sánchez, Leonardo E Valdivia, Miguel L Allende

**Affiliations:** 1 FONDAP Center for Genome Regulation, Facultad de Ciencias, Universidad de Chile, Casilla 653, Santiago, Chile; 2 Present address: Department of Cell and Developmental Biology, University College London, London, UK

**Keywords:** Lateral line nerve, Schwann cells, Neurectomy, Nerve regeneration

## Abstract

**Background:**

Peripheral nerve injuries can severely affect the way that animals perceive signals from the surrounding environment. While damage to peripheral axons generally has a better outcome than injuries to central nervous system axons, it is currently unknown how neurons re-establish their target innervations to recover function after injury, and how accessory cells contribute to this task. Here we use a simple technique to create reproducible and localized injury in the posterior lateral line (pLL) nerve of zebrafish and follow the fate of both neurons and Schwann cells.

**Results:**

Using pLL single axon labeling by transient transgene expression, as well as transplantation of glial precursor cells in zebrafish larvae, we individualize different components in this system and characterize their cellular behaviors during the regenerative process. Neurectomy is followed by loss of Schwann cell differentiation markers that is reverted after nerve regrowth. We show that reinnervation of lateral line hair cells in neuromasts during pLL nerve regeneration is a highly dynamic process with promiscuous yet non-random target recognition. Furthermore, Schwann cells are required for directional extension and fasciculation of the regenerating nerve. We provide evidence that these cells and regrowing axons are mutually dependant during early stages of nerve regeneration in the pLL. The role of ErbB signaling in this context is also explored.

**Conclusion:**

The accessibility of the pLL nerve and the availability of transgenic lines that label this structure and their synaptic targets provides an outstanding *in vivo* model to study the different events associated with axonal extension, target reinnervation, and the complex cellular interactions between glial cells and injured axons during nerve regeneration.

## Background

The nervous system of mammals reaches a high degree of complexity during development and, for the most part, progressively loses its ability to regenerate in adulthood. The loss of some cell types, such as central nervous system neurons and their projections are permanent after tissue damage [1,2]. In contrast, injuries to peripheral nerves have a better prognosis [3]. It is known from studies in the mouse that, after nerve injury, the distal portion of the nerve degenerates [4,5] and that, after about 1 week, the neurons start to regrow their axons from the proximal fragment [6,7]. However, the complete and successful recovery and re-establishment of neural connections depends on whether peripheral axons activate their intrinsic capability of regrowth through a complicated network of cellular and molecular components back to their target cells [3,8,9].

In the peripheral nervous system, Schwann cells contribute greatly to axonal regeneration after nerve injury [10]. In mammals, it has been shown that, following nerve injury, Schwann cells decrease the expression of myelin and the myelin sheath itself is lost, while there is an increase in the expression of transcription factors associated with an immature Schwann cell state [11-14]. Furthermore, during the first days post-injury, Schwann cells actively process and digest myelin debris in intracellular vacuoles and expose the extracellular myelin debris for engulfment by macrophages [15-17]. Furthermore, the dedifferentiated Schwann cells form unique columnar structures known as Bands of Büngner, along which regenerating axons can regrow [18-20]. Upon successful axonal regeneration, Schwann cells regain their contact with axons and undergo differentiation once again into myelinating cells [21]. Surprisingly, when Schwann cells are unable to dedifferentiate, they become less supportive of regeneration: myelin debris persists, neuronal death increases, and functional recovery is impaired [14,22].

Given that the recovery of a damaged sensory system represents an extremely complex goal for an organism, the mechanisms underlying this phenomenon are the object of intense study [23-25]. Recently, the lateral line of the zebrafish has emerged as a useful model for studying the interactions between peripheral axons and extrinsic cell types during development and regeneration [26-31]. The lateral line of zebrafish is a mechanosensory system able to detect and localize water movements around the body of the animal. It is comprised of two branches named the anterior and posterior lateral lines (aLL and pLL), which cover the entire body of the fish. The sensory information detected through this system allows to the fish to control different complex motor behaviors such as navigation, schooling rheotaxis, and predator avoidance [32-35]. The vast majority of the knowledge about this sensory system comes from the study of its posterior component. The pLL develops from a migratory primordium (prim I) that moves along the horizontal myoseptum from the head to the tip of the tail at embryonic stages [36]. During its journey, between 20 to 40 hours post fertilization (hpf), the primI deposits seven to eight prosensory units called neuromasts as well as interneuromastic cells that are located between them [37]. After deposition, each neuromast differentiates into a central cluster of mechanosensory hair cells surrounded by different types of accessory cells [36]. The interneuromastic cells remain in quiescent state until later during development when they proliferate to give rise to new neuromasts to shape the adult lateral line [37-39]. From 32 hpf to 6 days post fertilization (dpf), three to four secondary lateral line neuromasts are added between the pLL ganglion and the anus by a secondary migratory primordium (primII) [36,39,40].

The placodal field that originates the pLL primordium also gives rise to the pLL ganglion, where afferent sensory neurons are born. When the primI starts to migrate, neurons located in the pLL ganglion extend their axons such that they comigrate with the primordium and form a pioneer tract for subsequent axons to follow along the horizontal myoseptum [27,29,41]. In this way, they innervate the neuromast hair cells soon after neuromasts are deposited and these cells differentiate. In addition, sensory axons serve as a guiding trail for neural crest-derived glial cells that maintain the correct fasciculation of the nerve, these later differentiate into Schwann cells [28], reviewed in [42]. Therefore, the tripartite relationship between the migrating primordium, sensory axons and glia is a hallmark of the development of this system. Furthermore, the afferent bipolar pLL neurons project centrally to the hindbrain, where a somatotopic sensory map is established [27], reviewed in [36,42-45]. By 3 dpf, the neuromasts of the pLL and the entire basic behavioral circuit are functional.

Further evidence has added an additional degree of complexity to the innervation of the lateral line. The afferent neurons of the pLL ganglion are heterogeneous and have been classified into at least two types. The leader neurons (or type A) are the first to differentiate, are characterized by a large soma and are distributed throughout the entire ganglion [27,43,46]. The peripheral axonal projections of these neurons comigrate with the primordium and innervate the primary neuromasts [43,45]. The type B, later-born neurons, or followers, project their peripheral axons once primI has migrated a considerable distance [27]. These younger neurons are able to innervate primary or secondary neuromasts [40,42,43].

The pLL has been extensively used as a model for hair cell regeneration, which has begun to unravel the processes regulating tissue homeostasis in this system [47-51]. However, the pLL nerve is a relatively poorly explored system to address the issue of axon regeneration, although it retains a robust capability and fidelity to regrow upon axonal damage [30,31,37]. In this work we take advantage of the convenient superficial location of the pLL nerve, the availability of transgenic lines that label different cellular components of this system, and the stereotyped position of the target organs, to characterize the temporality and specificity of the different events elicited by axonal damage in zebrafish. Axonal regeneration upon damage has been demonstrated in the pLL, where complete neuromast reinnervation is seen 24 h after injury [30]. However it is not known if Schwann cells respond with similar dynamics and whether events that have been described in mammals (for example, dedifferentiation of Schwann cells) are conserved in this experimental system.

Here, we use a simple and inexpensive electric neurectomy method to study axonal regeneration in the pLL nerve of larval zebrafish [52]. When combined with single cell-labeling and cell transplantation experiments, this approach allowed us to monitor the cellular interactions between glial cells and axons during the regenerative process as well as target reinnervation *in vivo*. We found that neuromast reinnervation is a highly dynamic event with imprecise target recognition after regeneration. Despite this, sensory axons predominantly reinnervate neuromasts neighboring their original targets suggesting that the sensory field is likely to be restored. The growth and fasciculation of the regenerating nerve is coordinated by Schwann cells. Both regrowing axons and Schwann cells cooperate during early stages of nerve regeneration in the pLL and we provide evidence that ErbB signaling is important for this relationship. The superficial and accessible location of neurons and synaptic targets provides an attractive *in vivo* system to study the events related to axonal extension, target reinnervation, and cellular interactions between glia and regenerating axons.

## Results

### Reorganization of sensory innervation after pLL nerve regeneration

To better understand how the reconnection of a functional sensory system is established after peripheral nerve degeneration/regeneration, we took advantage of the simple anatomical organization of the larval posterior lateral line (pLL) in zebrafish. In this sensory system, the target organs, neuromasts, are located along the body surface in stereotyped positions [53].

We generated localized ablations of the pLL nerve in 3-day-old (3 dpf) larvae using electroablation, a technique recently developed in our lab [52]. This technique severs the nerve and also ablates the underlying Schwann cells; we carry out neurectomy halfway between the pLL ganglion and the first pLL neuromast (L1). We chose to carry out experiments in 3 to 5 dpf fish because, at this stage, the larvae are still highly transparent allowing us to distinguish and follow single neurons and their projections very easily. As larvae grow, transparency is reduced hindering single axon observation (Additional file 1; compare figure A vs. D, and A’ vs. C’). Furthermore, sensory cells in the pLL neuromasts have differentiated and the basic neural circuit in this system is functional at this stage.

Using electroablation, we have shown that pLL axon regeneration occurs with similar dynamics compared to two-photon ablation of the nerve [52]. In our previous studies we also demonstrated that the regenerated pLL axons arise from peripheral projections that grow from the axonal stumps of pre-existing neurons and not by replacement of injured neurons [30]. However, we ignored whether regeneration of individual axons innervate exactly the same sensory cells that were innervated by the original axon before axotomy.

**Figure 1:**
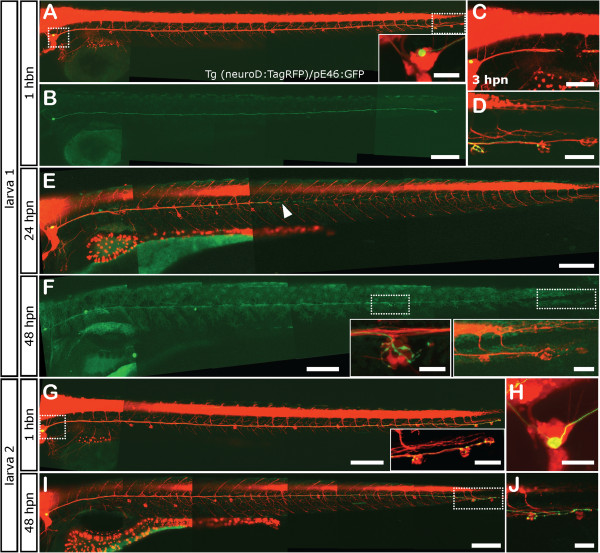
**Neuromast reinnervation after PLL nerve regeneration. ***Tg(neurod:TagRFP)* fish that have a red labeled pLL nerve were injected at the one cell stage with the *pE46:GFP* DNA construct and were selected if they displayed green fluorescence in a single sensory neuron in the pLL ganglion (inset in A). The innervation pattern of the single sensory neuron was recorded both before neurectomy and after regeneration of the axon. Two different examples of reorganization during pLL nerve regeneration are shown, referred to as larva 1 **(A-F)** and larva 2 **(G-J)**. Larva 1 shows a pLL ganglion neuron that innervates the terminal neuromasts before injury **(B, D)**. Neurectomy is carried out about 200 μm away from the ganglion severing all axons of the pLL nerve **(C)**. Twenty-four hours post neurectomy (hpn), the pLL nerve has regenerated about half way down the body of the larva **(E)**. At 48 hpn, the nerve has completely regenerated **(F)** altough the green-labeled axon now innervates a different neuromast, the L4 (small inset in **F**) and does not innervate its original targets, which are innervated by other neurons (large inset in **F)**. Larva 2 shows innervation of the terminal neurmasts before injury **(G, H)** and, after neurectomy and regeneration, the same neuromasts are reinnervated **(I, J)**. Scale: **A**, **B**, **E**, **F**, **G**, **I**: 200 μm; **C**: 100 μm; inset in **A**, **D**, larger inset in **F**, inset in **G**, **H**; small inset in **F**: 20 μm.

Thus, in order to determine the fidelity of this system upon nerve injury, we first stochastically labeled single pLL neurons by injection of *HuC:mem-TdTomato* or *pE46:GFP* DNA into transgenic *tg(neuroD:EGFP)* or *tg(neuroD:tagRFP)* embryos at the one-cell stage, respectively. We screened for transient transgenic embryos expressing TdTomato or GFP in single lateral line neurons at 3dpf. Selected larvae were imaged 1 h before neurectomy (hbn) to identify the neuromast(s) innervated by the labeled neuron. Afterwards, larvae were neurectomized using an electrical pulse between the pLL ganglion and the first neuromast (L1) and the larvae were left to recover at 28°C, as decribed before [52]. We analyzed the structure of both the axon and the nerve at 24 and 48 hours post neurectomy (hpn) (Figure 1).We found that axons displayed a variable reinnervation behavior during regeneration. In Figure 1 we show two different examples that are representative of the different situations encountered. Larva 1 shows a single neuron labeled by GFP that innervated the terminal-most neuromasts (L5-L7; Figure 1A-D). After neurectomy (Figure 1C), this neuron changed its sensory target once regeneration was achieved (48 hpn) innervating a different neuromast (L4). After regeneration, the neuromasts originally innervated by this neuron are now innervated by other neurons, labeled by RFP (insets in Figure 1F). The second example (larva 2) shows a neuron displaying a large soma innervating the terminal-most neuromasts. After neurectomy and regeneration, this cell extended its peripheral axons to the same targets (Figure 1G-J).

**Figure 2:**
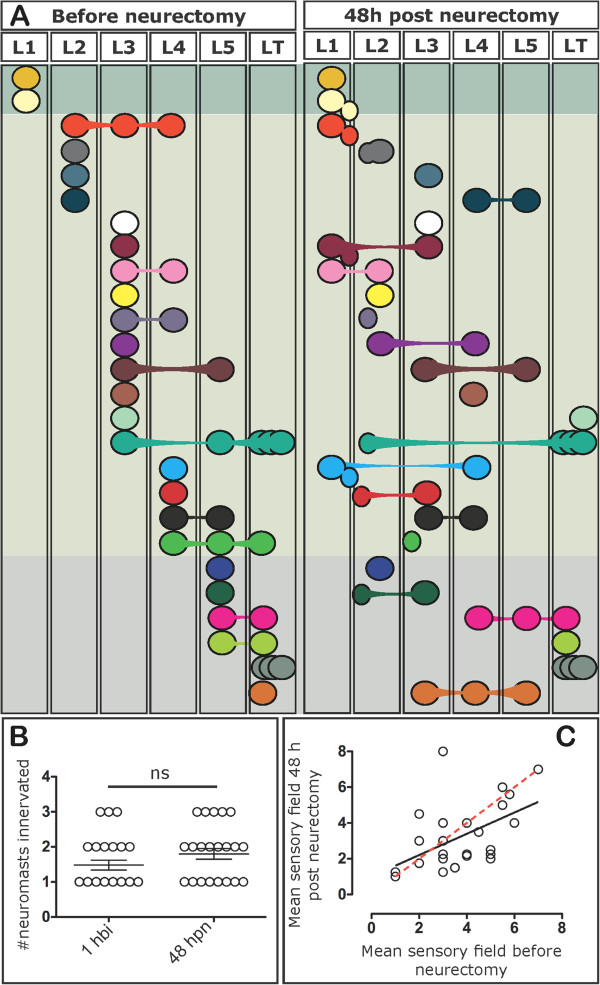
**Schematic representation of the pLL neuromast innervation patterns before and after nerve regeneration. (A)** The specific innervation of neuromasts (L1 to L5 or the terminal neuromasts, (LT) by single labeled pLL ganglion neurons was recorded before neurectomy and after nerve regeneration (48 hpn). Each neuron belongs to a different larva and is represented by a unique color in the diagram. The larger circles represent the innervated primary neuromasts whereas the small ovals represent secondary neuromasts. **(B)** The number of neuromasts innervated by single labeled neurons was recorded before (1 hbi) and 48 hpn. The average number of neuromasts innervated is not significantly different between both samples (*P* <0.05). **(C)** The pattern of pLL neuromast innervation before neurectomy and after regeneration was compared by calculating the ‘mean sensory field’ of individual neurons in both conditions (calculated from data shown in panel A; see Methods). A perfect fit (reinnervation of the exact same targets after regeneration) is shown as a dashed red line, and the actual data is the solid black line (significance of fit: *P* = 0.009). Statistically (*P* = 0.0618), both slopes are equal.

Using the strategy described above, we examined individual axons in DNA injected larvae and recorded which neuromasts were innervated pre vs. post neurectomy (Figure 2). We considered only afferent neurons whose peripheral projections were recognizable continuously throughout the experiment in order to ensure that we were always evaluating the behavior of the same neuron (see Additional file 1).

In intact 3 dpf fish (pre-neurectomy), single neurons were seen to innervate more than one neuromast, as reported previously by Sarrazin *et al.*[40]. Interestingly, the average number of neuromasts innervated by single neurons did not change significantly comparing before and after neurectomy (Figure 2B; Additional file 2). Furthermore, before neurectomy, at 3 dpf, when the primII is still migrating, the labeled neurons exclusively innervated neuromasts of the primary lateral line whereas, after regeneration, primary and secondary neuromasts could both be innervated by the same regenerated neuron.

When we examined the specificity of the reinnervation pattern compared to pre-neurectomy, there was a high degree of variation. Only about one half of the neurons reinnervated the same neuromast (13 out of 27) (Figure 2A). Nonetheless, we wished to determine whether the anteroposterior innervation pattern was preserved after regeneration of pLL axons, as this system manifests robust somatotopy when it is initially formed [27,43]. As many axons innervate more than one neuromast, we calculated the median neuromast position (L1 to L5 or LT) innervated by a neuron both before and after neurectomy and we generated a plot in which the axes represent the pre- and post-neurectomy average innervated neuromast (Figure 2C). The graphed data show that there is a significant positive correlation between both conditions and the slope of the line of best fit is not significantly different from a perfect correlation (Figure 2C). Therefore, while there is a degree of promiscuity and original target organs are not reacquired with absolute fidelity, the overall anteroposterior sensory map is reproduced after regeneration of pLL nerve axons. Furthermore, fish that have suffered neurectomy are indistinguishable from control larvae in terms of swimming behavior after 2 days post neurectomy (dpn) and 100% of neurectomized larvae inflate the swim bladder at 4 dpf as controls do (data not shown).

### Schwann cells and regrowing axons cooperate during the first stages of nerve regeneration in the pLL

In addition to the high regenerative capacity of the pLL nerve, the regrowing axons always follow the horizontal myoseptum [30]. In our hands, the nerve is indistinguishable from the original in terms of structure and trajectory after 48 hpn. In some cases, we have observed a minor defasciculation of the nerve within the area where electroablation created a gap in the tissue.

**Figure 3:**
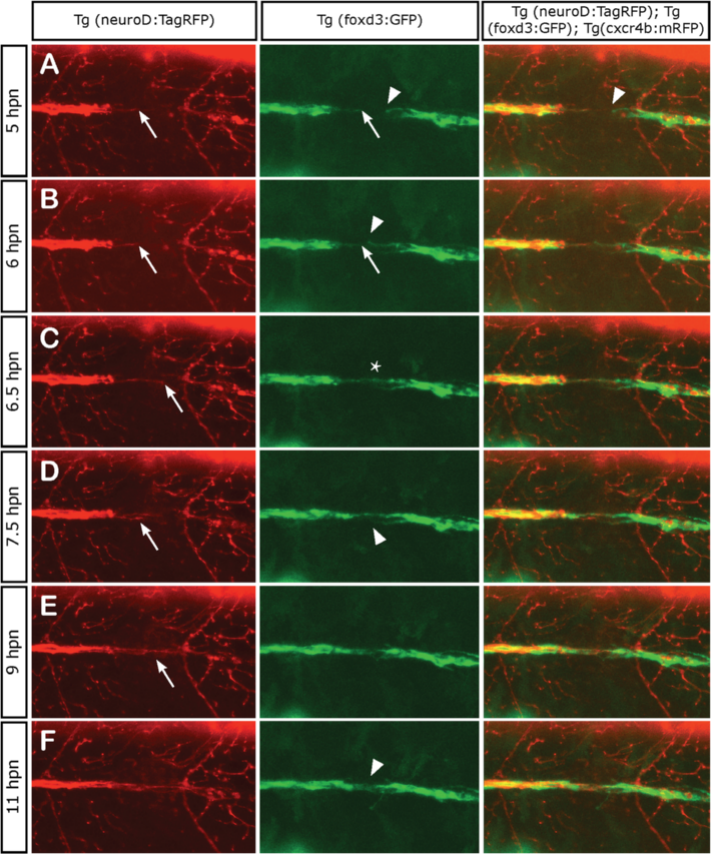
**Axonal and Schwann cell behaviors at the point of neurectomy.** Three days post fertilization transgenic *Tg(neurod:TagRFP;foxd3:GFP)* zebrafish larvae were neurectomized and imaged from 5 hpn to 11 hpn every 2 min following complete nerve transection (axons in red and Schwann cells in green). In all panels, the arrows show the behavior of axons and Schwann cells proximal to the gap whereas the arrowhead shows the behavior of Schwann cells distal to the gap. **(A, B)** A few hours after neurectomy, distal (denervated) Schwann cells extend their processes within the gap and show an exploratory behavior, whereas proximal Schwann cells are less motile. **(C)** At 6.5 hpn, the regrowing axons have contacted distal Schwann cells and have formed a bridge across the gap (asterisk). **(D, E)** After 7 hpn, the axons complete the crossing of the gap; often, the first axon to navigate the gap stops growing and another axon takes the lead. **(F)** After 11 hpn, the regrowing nerve has grown past the gap enabling the reconnection between proximal and distal Schwann cells in 100% of neurectomized larvae.

Given that a failure in axonal regeneration usually arises as a result of sprouting of damaged axons that must break through the injury site without guidance cues, we took advantage of our experimental setup of neurectomy, which not only sections the nerve but also leads to local death of Schwann cells within a diameter of 80 to 100 μm, to examine this process. We analyzed the behavior and interaction between glial cells and damaged axons at the injury site during the first hours of axon regeneration *in vivo*. We neurectomized 3-day-old *Tg(neurod:TagRFP;foxd3:GFP)* double transgenic larvae that exhibit differentially labeled pLL axons and neural crest derived Schwann cells [28,54]. We carried out time-lapse recording beginning 5 h after nerve transection, a time in which removal of axonal debris has concluded and nerve elongation begins (see Additional file 3). We noticed a difference in the behavior of Schwann cells located proximal to the gap (still in contact with the axonal stump) in comparison to the denervated Schwann cells located distal to the injury site. While distal Schwann cells showed increased motility and exploratory behavior with process extensions (Figure 3A and B arrowhead), the proximal population extended processes only following pioneering axon extension through the gap (Figure 3A and B, arrow). The exploratory behavior of the regrowing axons and the protrusion and movements of denervated Schwann cells, contributed to form a bridge between both elements in such way that the axon regrowth was guided across the gap permitting the reconnection between Schwann cells located on both sides (asterisk in Figure 3C).

An interesting observation, derived from time-lapse images, is that the regenerating axons did not grow at a constant velocity over time. We found that, after a leading axon contacted Schwann cells, in many cases it stopped growing after a few microns. During such pausing, another axon took the lead (Figure 3D, arrow). Thus, the axons crossed the gap in a sort of competition, a phenomenon observed in other contexts [55-57]. For this reason it was difficult to refer to a specific neuron as a leader or follower when examining growth through the gap. By 11 hpn the nerve had entirely bridged the gap enabling the reconnection between Schwann cells of both sides in 100% of neurectomized larvae. Finally, only when the gap was crossed by the axons, Schwann cells migrate to seal the injury site and generate a continuous line of Schwann cells.

Our data confirm previous reports showing a close relationship between regenerating axons and Schwann cells [30]. Interestingly, it also suggests that even if the glia provides a scaffold for nerve re-elongation, sealing a gap in the glial cell continuum depends on the nerve itself.

### Schwann cell response to peripheral nerve injury

In mammals, diverse cellular behaviors (including dedifferentiation, proliferation, further differentiation, cell death, and/or myelination) have been observed after injury [3,14,58-60]. Given that, in zebrafish, regeneration is much faster than in mammals, we were interested in characterizing the Schwann cell response, if any, triggered by nerve injury.

**Figure 4:**
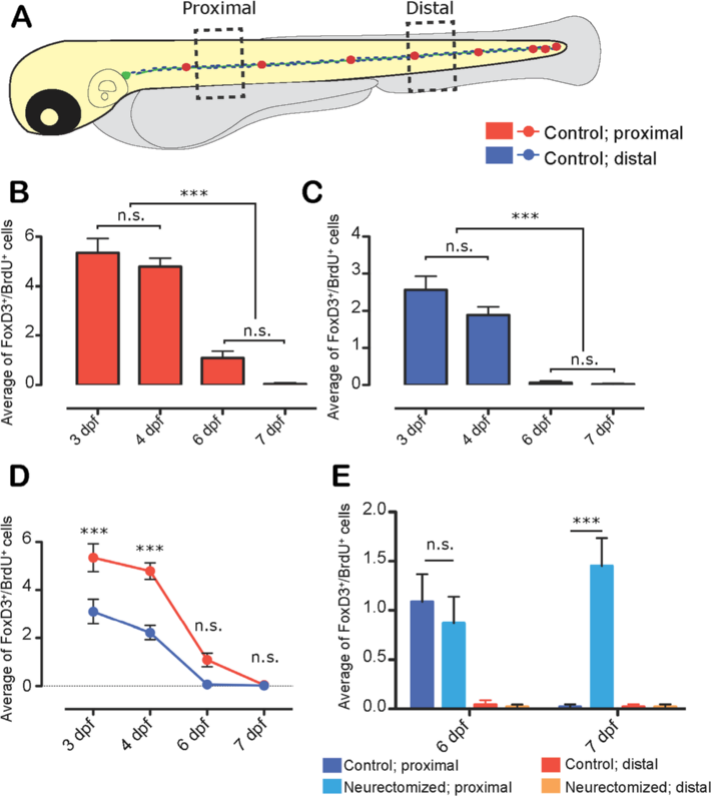
**Quantification of proliferative Schwann cells in control vs. neurectomized larvae. (A)** Schematic representation of the two areas of the larvae in which the number of proliferative (BrdU incorporating) Schwann cells was determined. The proximal area spans between the sixth to the eighth somites (two somites behind the neurectomy point) and the distal area begins two somites posterior to the anus. Proliferation was measured at different time points by BrdU incorporation in *tg(foxd3:GFP)* larvae (see Methods). **(B, C)** Schwann cells are actively proliferating from 3 to 4 dpf in control larvae. By day 6 however, there is a significant reduction in the number of proliferative cells in both areas analyzed, and by day 7 there is essentially no BrdU labeled Schwann cells. In **(D)**, data from panels **B** and **C** is graphed to compare proliferation in both areas. **(E)** The proliferative response of Schwann cells to denervation was evaluated at days 6 and 7 post neurectomy, when proliferation in these cells has normally ceased. *tg(foxd3:GFP)* transgenic larvae were neurectomized at 5 dpf and the number of GFP+/BrdU + cells was quantified in control and neurectomized larvae at 6 dpf (24 hpn) and 7 dpf (48 hpn). The number of proliferating Schwann cells was different between control and neurectomized fish at 48 hpn in the proximal area. n.s: non-significant, **P <0.01, ***P <0.001.

To examine cell proliferation in these cells, we began by assessing Bromodeoxyuridine (BrdU) incorporation from 72 hpf to 7 dpf in *tg(foxd3:GFP)* untreated larvae (Figure 4). We carried out the analysis in two areas: in the trunk (proximal), near the point where we routinely carry out neurectomy, and in the tail (distal) (Figure 4A, see methods). In both areas analyzed, Schwann cells showed the same behavior. Between 72 hpf to 4 dpf, these cells proliferate at approximately the same rate, but this rate drops to zero around 6 to 7 dpf, coinciding with the start of mielynation [61] (Figure 4B and C). The proliferative rate of Schwann cells until 4dpf was always higher in the proximal region compared to the distal region (Figure 4D).Since Schwann cells are actively proliferating until 4 dpf, we decided to carry out neurectomy at 5 dpf and follow proliferation until 7 dpf, so that the normal proliferation in these cells does not interfere with our analysis. Neurectomized and control larvae were given a BrdU pulse at different time windows (24 to 27 hpn; and 48 to 51 hpn, see Methods). The incubated larvae were then fixed immediately after BrdU incubation. Whereas in control larvae there is no proliferation at 7 dpf, the Schwann cells of neurectomized larvae continued to proliferate, albeit only in the proximal region, at this stage (48 hpn) (Figure 4E).

**Figure 5:**
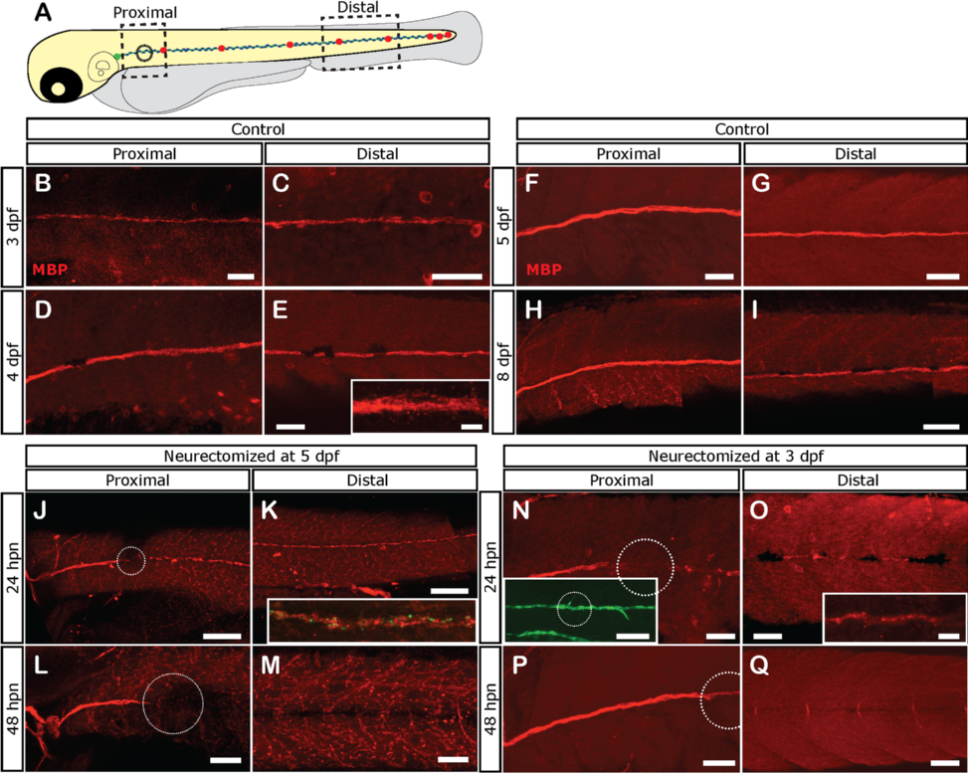
**Loss of Schwann cell differentiation markers after denervation.** Tg(*foxD3:GFP)* larvae were left untreated or neurectomized and then fixed at different times and processed for immunostaining with anti-Myelin Basic Protein (MBP; labeled in red). **(A)** The expression of MBP was evaluated in a proximal and distal area with respect to the point of neurectomy (black circle). **(B-I)** In control larvae. MBP is expressed at proximal and distal levels at 3 dpf and increases as the larva ages. **(J-M)** Expression of MBP becomes absent after neurectomy in denervated Schwann cells. Five-day-old *tg(NeuroD:GFP)* larvae were neurectomized and were fixed and processed for immunostaining with anti-MBP 1 **(J, K)** or 2 days **(L, M)** after neurectomy. Nerve degeneration (green label in inset in **K**) is followed by fragmentation of MBP expression in Schwann cells distal to the injury point (white dotted circle) at 24 hpn (**J**, **K**, inset in **K** shows both channels). At 48 hpn **(L, M)**, MBP expression has disappeared distal to the injury site. **(N-Q)** Neurectomy at earlier stages, when MBP becomes expressed (at 3 pdf), showed the same results. In this case, Tg(*foxD3:GFP)* larvae were used to follow Schwann cells; note that despite fragmentation and loss of the MBP label, Schwann cells still remain viable as revealed by GFP expression. Scale bars: **J**, **K**, inset in **N**: 100 μm; **B-I**, **L-Q**, inset in **E**, inset in **O**: 50 μm.

Since the differentiation status of Schwann cells has been seen to change after neurectomy in mammals, we analyzed the expression of Myelin Basic Protein (MBP) in control and neurectomized zebrafish larvae. MBP is a terminal differentiation marker of myelinating glia in the central and peripheral nervous systems [61]. At 3 dpf, it was possible to observe incipient expression of MBP in the lateral line at proximal and distal levels, suggesting that Schwann cells associated with this nerve have begun myelination at this stage (Figure 5B, C). The expression of this marker increases progressively over time from 4 dpf to 8 dpf in untreated larvae (Figure 5D-I). Larvae that were neurectomized at either 3 dpf or 5 dpf, showed fragmentation of the MBP label beyond the injury site by 24 hpn (Figure 5J. K, N, and O) and a total loss of this marker from the injury site to caudal regions after 48 hpn (Figure 5L, M, P, and Q). Interestingly, this rapid loss of MBP in Schwann cells of neurectomized fish happens despite the fact that Schwann cells continue to express GFP driven by the *foxd3* promoter (see Figure 3) and display motile behavior that allows reconnection of the gap created at neurectomy (inset in Figure 5N). In larvae that were neurectomized at 3 dpf, 2 days after neurectomy (48 hpn), an anteroposterior wave of MBP reappearance was observed (Additional file 4A) that reached the tip of the tail after 5 dpn, even though the label was considerably lower in neurectomized larvae when compared to controls (Additional file 4, compare B vs. C).

**Figure 6:**
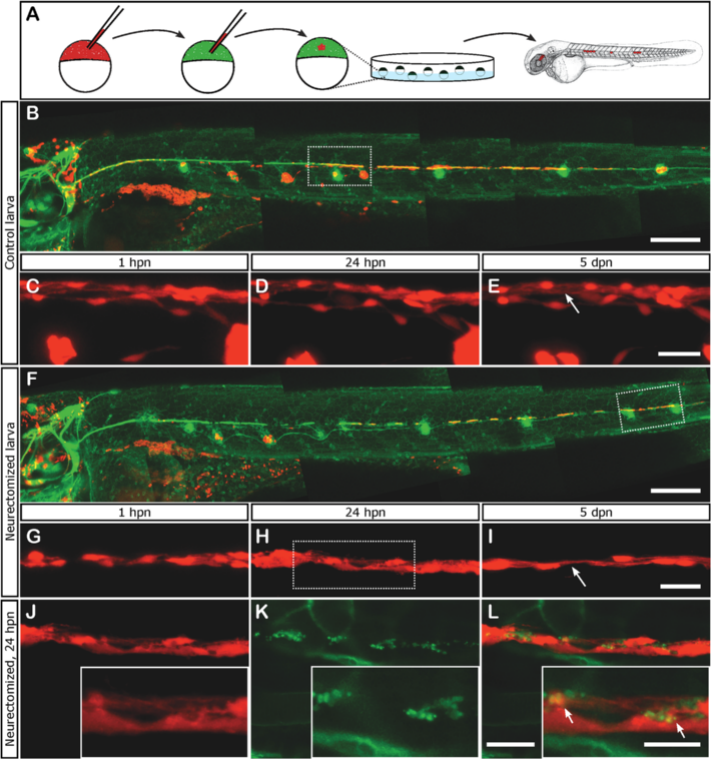
**Fate of Schwann cells after neurectomy. (A)** Schematic representation of the transplantation scheme used to obtain mosaic labeling of Schwann cells. Briefly, 10 to 20 donor cells of *Tg(Ubi:zebrabow:cherry)* blastula stage embryos were aspirated and transplanted into *Tg(8.0cldnb:lynEGFP)* host embryos of the same stage. The transplanted embryos were screened for the presence of red fluorescent donor cells in the pLL at 48 hpf. **(B)** Distribution and morphology of transplanted Schwann cells in a mock-neurectomized larva (control). The rectangle indicates the region examined in the same fish at different timepoints.** (C-E)** Schwann cells were examined from 1 hpn **(C)** to 5 dpn **(E)**; the cells show the same distribution and general morphology through time; however, at 5 dpn (which corresponds to 8 dpf) the cell extensions form a tubular structure typical of myelinating Schwann cells (arrow). **(F)** A transplanted larva that was neurectomized; the rectangle indicates the region detailed in **G-I**. **(G-I)** Organization and morphology of Schwann cells over time, from 1 hpn **(G)** to 5 dpn **(I)**. At 24 hpn **(H)** the cells exhibited vacuoles and lost their typical bipolar morphology. After 5 dpn (8 dpf) Schwann cell numbers appear reduced and the cells show thinner processes and fail to form the extensions seen in control fish (compare **E** to **I** arrows). The rectangle in **H** shows the region expanded in **J-L. (J-L)** One day after neurectomy, Schwann cells appear to engulf debris from damaged axons (arrows in **L**). Scale bars: **B**, **F**: 200 μm; **C-E**, **G-I**: 25 μm; **J-L**: 20 μm; insets in **J-L**: 10 μm.

A consistent observation in fish neurectomized at both 5 dpf and 3 dpf was that GFP levels in *tg(foxd3:GFP)* larvae diminished caudal to the neurectomy point even though the axons had regenerated to the tip of the tail after 48 h (Additional file 5, compare 5B vs. 5C, and 5E vs. 5 F). This lower level of GFP label in the line of Schwann cells that accompany the pLL nerve persisted for several days after neurectomy (data not shown). The changes detected in Schwann cells after neurectomy (extension of the proliferative phase, loss of MBP, and diminishing GFP expression), prompted us to look for additional signs that could be indicative of a reversion of the differentiated state such as changes in cell shape or cell size. GFP expression in *tg(foxd3:GFP)* larvae did not allow us to observe individual cells, so we generated mosaic animals by transplanting cells from *Tg(ubi:zebrabow:cherry)* embryos, which stably and permanently express red fluorescent protein, into *Tg(8.0cldnb:lynEGFP)* embryos as hosts, in which all the cells derived of the primordium and the nerve of the lateral line express a membrane bound GFP [62]. In transplanted fish at 48 hpf we screened for the presence of red-labeled donor cells in the myoseptum, where Schwann cells are expected to be (Figure 6A). These patches of genetically labeled glial cells allowed us to analyze their number, distribution, and morphological changes through time after denervation, with a higher level of resolution. We imaged transplanted Schwann cells from 1 hpn to 5 dpn at low magnification (20×) and we also imaged selected regions with a 40× objective. In control larvae, the distribution and the number of Schwann cells remained essentially unchanged from 3 dpf to 8 dpf, both at proximal and distal positions, although a small increase in the number of cells over time can be seen, especially in the anteriormost portion of the glial chain of cells (Figure 6B-E). From 4 dpf to 8 dpf, cell processes extended, suggesting the formation of the tubular structure that is typical of myelinating Schwann cells (Figure 6E, arrow). In neurectomized larvae, the morphology and number of Schwann cells changes compared to control fish. At 1 hpn the cells showed the same bipolar morphology as controls did (Figure 6G) but, after 24 hpn, the cells appeared vacuolated (Figure 6H). Figures 6J-L show higher magnification images of Schwann cells (in red, Figure 6J) and the degenerating nerve (in green, Figure 6K) at this stage after neurectomy. Combination of both images allows the detection of GFP labeled membranes closely associated with Schwann cells, suggesting that axonal debris may be engulfed by Schwann cells *in vivo* (Figure 6L). Later, at 5 dpn, (8 dpf), neurectomized larvae displayed abnormal Schwann cells, both in shape and in number, compared to controls (compare Figure 6E to 6I). The Schwann cells display a bipolar morphology, more typical of the more undifferentiated state. Furthermore, at this stage, Schwann cells have not yet formed the tubular structures seen in control larvae (compare Figure 6I with 6E, arrow).

These data lead us to conclude that the response of Schwann cells after a peripheral nerve injury is conserved, at least in part, between zebrafish and mammals. In fish, Schwann cells lacking a peripheral nerve respond very quickly to loss of the nerve and lose differentiation markers within the first day after neurectomy.

### Schwann cells are required for correct pLL nerve regeneration

Schwann cells wrap the pLL nerve and have been shown to carry out phagocytosis of debris during axonal degeneration after nerve sectioning [5,16]; reviewed in [63]. Given that our results support conservation of Schwann cell responses to neurectomy between fish and mammals, we next asked whether Schwann cells play a role during pLL nerve regeneration. To study this relationship, we analyzed the behavior of damaged and regenerating pLL axons in absence of these glial cells in zebrafish larvae.

**Figure 7:**
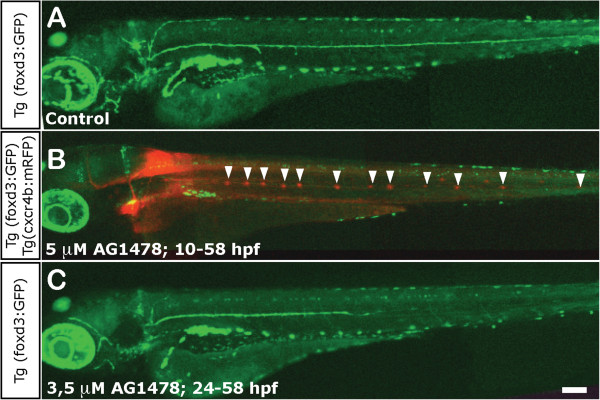
**Impairment of Schwann cell migration following treatment with AG1478. (A)** Normal distribution of Schwann cells in a 3 dpf control foxD3:GFP larva. **(B)** Total absence of Schwann cells in 3 dpf foxD3:GFP/Cxcr4:mCherry larvae treated with 5 μM AG1478 from 10 to 58 hpf (in this double transgenic, neuromasts are labeled in red fluorescence and Schwann cells in green). The supernumerary neuromasts that form in the absence of glial cells are indicated by arrowheads. **(C)** Partial absence of Schwann cells in 3 dpf foxD3:GFP larvae treated with 3.5 μM AG1478 from 24 to 58 hpf. With this treatement the Schwann cells migrate as far as the posterior end of the trunk (±two somites), but not into the tail at 3 dpf. Scale: **A-C**: 100 μm.

First, we interfered with migration of Schwann cell precursors during their development by incubating larvae with AG1478. This drug effectively depletes peripheral nerves of Schwann cells if administered prior to the beginning of their migration along the growing pLL nerve [61]. Incubation of embryos between 10 hpf and 58 hpf with 5 μM AG1478 completely eliminated all Schwann cells, as expected (Figure 7A). We confirmed that Schwann cells were effectively absent by the appearance of supernumerary neuromasts, as has been reported [38] (Figure 7B, arrowheads). Additionally, incubation of the larvae with 3.5 μM AG1478 between 24 hpf and 58 hpf, allowed normal development of Schwann cells as far as the posterior trunk but prevented their development in the tail (Figure 7C). Twelve hours before neurectomy, we washed out the inhibitor in order to avoid any interference of the drug on pLL nerve regeneration.

**Figure 8:**
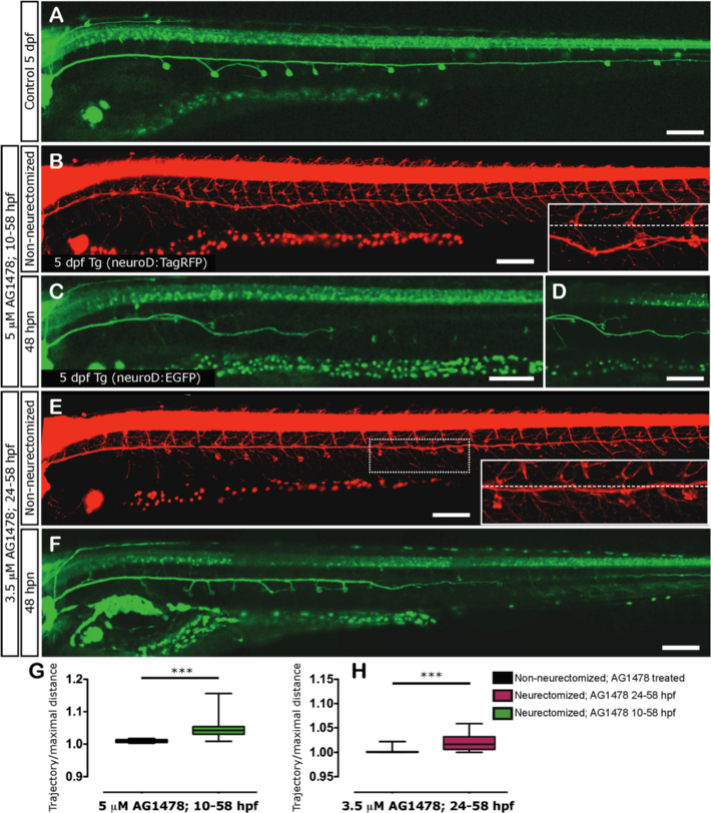
**pLL nerve regeneration in larvae totally or partially lacking Schwann cells.** Transgenic tg*(neuroD:EGFP)* or tg*(NeuroD:TagRFP)* were used to evaluate the impact of Schwann cell absence on pLL nerve regeneration. **(A)** After 48 hpn, the regrowing nerve of control but neurectomized larvae reaches the tip of the tail. **(B-F)** Transgenic larvae were treated with AG1478 following two treatments: 5 μM AG1478 from 10 to 58 hpf **(B-D)** and 3.5 μM AG1478 from 24 to 58 hpf **(E, F)**. In both cases, the drug was washed out at 58 hpf and the larvae were maintained in E3 medium. At 3 dpf, larvae of each treatment were separated into two groups: non-neurectomized AG-treated controls and neurectomized AG-treated larvae. Note that the nerve of control (and treated) larvae deviates from the myoseptum (dotted line) in both treatments **(B, E)** but deviation is more severe in the longer treatment; insets show larger magnification. In parallel, the pLL nerve of neurectomized larvae fails to regenerate properly as it defasciculates and wanders outside of the horizontal myoseptum **(C, D, F)**, and fails to reach the tip of the tail at 48 hpn under these conditions. **(G,H)** At 5 dpf we quantified and compared the winding index of the nerve in neurectomized vs. non-neurectomized larvae. In both treatments, the pLL axons of neurectomized larvae have an increased winding index in comparison with their respective controls. ***P <0.001. Scale: **A-F**: 100 μm.

At 3 dpf we neurectomized larvae treated previously with AG1478 and evaluated nerve regeneration after 48 hpn (Figure 8). Neurectomized larvae treated with 1% DMSO regenerated completely after 48 hpn and the nerve was indistinguishable from non-injuried larvae (Figure 8A). However, in larvae lacking Schwann cells, the regenerating axons of the pLL nerve failed to reach the tip of the tail at 48 hpn (Figure 8C). Axons in these fish displayed an explorative behavior, with intense growth and retraction dynamics in many directions outside the original path along the myoseptum (see Additional file 6). In some cases, the axons regrew a few microns and, after a short period of elongation, they retracted and stopped growth for long periods of time (Additional file 7). This erratic behavior causes defasciculation and ventral or dorsal diversion of nerve fibers all of which result in a severe impairments on axonal elongation along the horizontal myoseptum. Importantly, while these axons are extending, they do so at the same velocity displayed by regenerating axons in untreated axotomized larvae (approximately 0.8 μm/min, data not shown).

We next carried out partial inhibition of Schwann cell development, which resulted in a lack of these cells posterior to the anus. The axons of larvae treated in this fashion regrew at first as in control larvae, tightly fasciculated and associated with the Schwann cells present in the myoseptum, but after 24 hpn, the nerve stopped growing and was unable to reach the tip of the tail (Figure 8F). The behavior of the pioneering nerve axons once they enter the territory lacking Schwann cells was recorded and is shown as a time lapse movie (Additional file 8).

As we observed evidence of defasciculation and diversion of axons in pLL nerves growing without Schwann cells, we calculated the winding index of the nerve under the different conditions tested (see methods). We found a significant increase in the winding index in neurectomized larvae incubated with AG1478 with respect to control larvae (non-neurectomized) that were treated in parallel with AG1478 under the two regimens tested (Figure 8C vs. B, F vs. E; quantitation in 8G, H). This indicates that Schwann cells are indeed required for the proper fasciculation and pathfinding of regenerating axons towards their target cells but are not necessary for axonal elongation, supporting our previous data. As a final observation made in AG1478 treated fish, Schwann cells of non-neurectomized larvae always remained restricted to the myoseptum, whereas in neurectomized fish, Schwann cells could often be found at ectopic positions as they migrated following the aberrant trajectories of regenerating axons (Additional file 9). This result also provides evidence to indicate effective washout of the AG1478 inhibitor (as Schwann cells continue to develop and migrate) and it shows that the main cause of defasciculation is loss of Schwann cells and not a direct effect of the drug on axons. It also shows that Schwann cells are not able to position themselves independently of axons.

**Figure 9:**
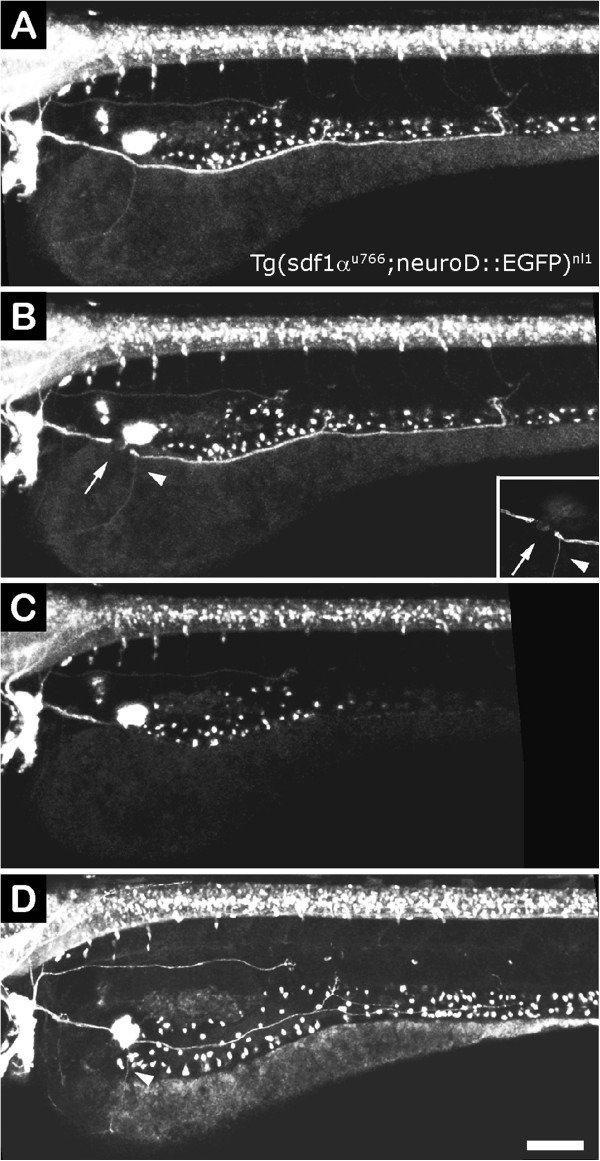
**pLL nerve regeneration in *sdf1 *mutant larvae. (A)** A 3 dpf *sdf1* mutant larva (also transgenic for neuroD:EGFP) shows a severely altered pLL due to failures in primordium migration during early development. The nerve is located over the yolk rather than along the horizontal myoseptum. **(B)** The larva was imaged immediately after neurectomy; the point of neurectomy is indicated by an arrow. Arrowhead points to an aberrant branch of the nerve that migrated towards the ventral yolk. **(C)** A few hours after neurectomy, the distal nerve has degenerated. **(D)** Twenty-four hours post neurectomy, the nerve has regenerated and follows exactly the same route as that of the original nerve, including the aberrant branch indicated in **B** (arrowhead). Scale: **A-D**: 100 μm.

We wished to provide further confirmation of this interdependence between Schwann cells and regrowing axons by carrying out regeneration experiments in the *sdf1a* mutant, in which the pLL primordium migrates aberrantly during development along the ventral body or yolk sac, misguiding the axons of the pLL nerve (C. Mardones, unpublished results). In the mutants, glial cells co-migrate with the ectopically growing nerve and are present in all the nerve branches that defasciculate to innervate nearby neuromasts (not shown). In order to neurectomize the nerve in mutant animals, we crossed the *sdf1a*^*u766*^ mutants into the *tg(neuroD:EGFP)* background, which labels the pLL axons [30,54]. At 3 dpf, *sdf1a* mutant nerves are observed to follow different pathways originating at the ganglion (Figure 9). After 24 hpn in mutant fish, the regrowing axons strictly follow the aberrant route established during development, maintaining close contact with the chain of glial cells that lie along the way and reproducing the exact pattern of nerve branching observed after initial development of the tract (Figure 9B and D, arrowhead).

Together, these results suggest that Schwann cells provide an essential role in guidance of regenerating pLL axons and, in turn, that pLL axons serve as a substrate for Schwann cell precursor migration during embryonic stages and during peripheral nerve regeneration.

### ErbB signaling is involved in axonal regeneration

**Figure 10:**
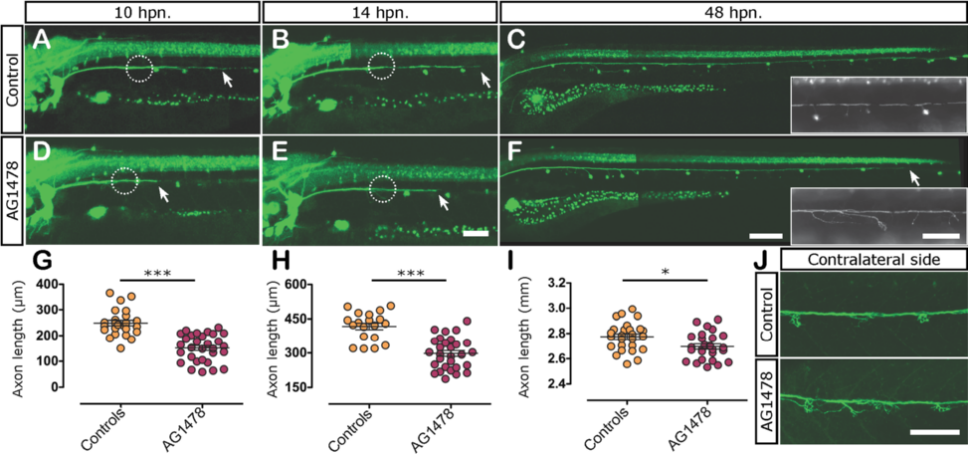
**Role of ErbB signaling in axonal regeneration.** Three days post fertilization larvae were neurectomized (circles indicate position of neurectomy) after incubation with 0.05% of DMSO (controls) or 5 μM of AG1478 from 60 hpf to 5 dpf (48 hpn); this treatment does not affect Schwann cell survival but blocks ErbB signaling during the regeneration phase. Nerve regeneration in control **(A-C)** and drug treated larvae **(D-F)** was evaluated after 10 hpn, 14 hpn, and 48 hpn. The insets in **C** and **F** show the defasciculation of the regenerated nerve in treated larvae in comparison to controls. **(G-I)** Quantitative analysis of axonal extension after 10 hpn **(G)**, 14 hpn **(H)**, and 48 hpn **(I)** in control and AG1478 treated larvae. *P <0.05; ***P <0.001. **(J)** In 30% of ErbB inhibitor treated larvae, the non-neurectomized contralateral nerve appears defasciculated in comparison to the control nerve. Scale bar: **C**, **F**: 200 μm; **A**, **B**, **D**, **E**, **J**; insets in **C** and **F**: 100 μm.

It has been recently shown that peripheral nerve injury causes an increase of ErbB receptor levels in the dorsal root ganglion after nerve injury [64]. To explore the requirements for ErbB signaling on axonal regeneration we performed pharmacological interference on this pathway using AG1478, after Schwann cell migration along the pLL nerve is complete (at 60 hpf in 5 μM of AG1478). We neurectomized the pLL at 3 dpf as before and we evaluated nerve regeneration in AG1478 treated and control larvae at different time points between 10 and 48 hpn. During the regenerative process, we maintained the neurectomized larvae in control or AG1478 solution until 5 dpf (48 hpn), once regeneration was complete.In control larvae, the regrowing axons traversed the gap left by pLL nerve injury between 6 and 9 hpn. The treatment with AG1478 caused a significant delay in the onset of regeneration as was evident when comparing treated to control fish at 10 hpn (compare Figure 10A vs. D and G) or at 14 hpn (Figure 10B vs. E; H). At 48 hpn, the nerve of the control larvae reached the tip of the tail in 100% of neurectomized control larvae, whereas neurectomized treated larvae were still regenerating their axons (Figure 10C vs. F; I). Although the treatment produced a delay in the onset of the regeneration process, it did not significantly affect the velocity of axonal elongation once the gap was crossed. In fact, control and treated larvae extended axons at a similar speed of approximately 0.65 μm/min on average from 10 to 14 hpn (not shown).

Given that the onset of regeneration was delayed with no impact on the subsequent nerve growth velocity, we suggest that the communication between axons and Schwann cells vía ErbB receptors could play a role in the early phases of pLL nerve regeneration, where Schwann cells and axons cooperate to cross the lesion site.

## Discussion

The functional re-assembly of a sensory system after damage represents a remarkable challenge that allows individuals to respond to environmental stimuli after an injury and, thus, survive. In the present study, we explored how a sensory system is arranged after nerve injury and characterized the interactions between axons and Schwann cells during pLL nerve regeneration in larval zebrafish. Using a simple set up for damaging this axon bundle in transgenic fish, in combination with single-cell labeling and cell transplantation, we have begun to dissect the influence of cell-cell interactions as well as the roles of specific molecular players in this process.

The pLL nerve of the zebrafish has been previously used as a model to for axonal regeneration and cell-cell interactions during this process [30,65]. Using two-photon laser ablation, Villegas *et al.*[30] showed that the pLL nerve has the capacity to regenerate after neurectomy becoming indistinguishable to that of control larvae after 24 hpn. In our hands, using electroablation, the nerve regenerates in about 35 hpn, a difference most likely due to the experimental protocols used in each case. Importantly, electroablation produces more extensive tissue damage and, thus, affects not only the peripheral fibers, but also Schwann cells and local muscle fibers localized at the neurectomy point, some of which die after the pulse see [52]. As an expected consequence, this methodology produces a much more significant inflammatory response as well [52], in comparison to that seen in previous studies [30,65,66]. It has been reported that tissue damage induces local release of hydrogen peroxide that can act as a diffusible signal modulating the regenerative response and the recruitment of immune cells to the wound margin [67]. This phenomenon clearly does not happen after single cell or single axon ablation with a focused laser [66]. Thus, we propose that damaging the pLL nerve using electroablation provides a more realistic approximation to a mechanical injury and resembles more accurately the types of scenarios encountered by individuals outside of a laboratory setting.

During regeneration of the pLL nerve, afferent neurons must re-establish the sensory connections with their targets in a fashion that, mechanistically, cannot recapitulate the developmental history of the system. By single neuron labeling we were able to recognize two previously described types of neurons [43-45]: older neurons with big somata (Figure 1 G-H), which correspond to leader neurons, and younger neurons called followers (Figure 1A-E). This could indicate that the pLL has not been refined yet, and that the axons of the leader neurons could be used as a scaffold for the extension of the younger axons. In several systems, it has been shown that leader neurons can undergo apoptosis once the follower ones have grown [68]. However, although suggested [43], this has not been convincingly demonstrated in the pLL. In our single-cell labeling experiments the possibility that regrowing axons arose from new born cells was excluded as we considered only neurons whose peripheral projections proximal to the stump (that are preserved after neurectomy) were recognizable through time (Additional file 1). We discarded larvae in which labeled cells disappeared at 3 dpf or if they were visible before neurectomy but not after, at 5 dpf (data not shown). While this may be due to a normal apoptotic event, we did not investigate this issue further.

Our data also show that, in the pLL, some of the leader neurons are capable of regenerating and reinnervating neuromasts, suggesting that these neurons play a role until later stages (Figure 1G and H). In fact, Sato *et al.*[43] revealed the presence of such neurons innervating the terminal neuromasts at 6 dpf. We do not know whether the presence of leader neurons is related to the regenerative capacity of pLL nerve. However, given that we see that reinnervation of the terminal neuromasts is more consistent and precise than reinnervation of other neuromasts (see below), we speculate that they can serve as a wiring scaffold for younger/follower axons, setting up the conditions for axon-axon communication and proper extension along the myoseptum in the absence of other guidance cues, such as those provided by the primordium.

Once regeneration has occurred, we found that innervation of neuromasts by pLL axons is dynamically reorganized after our injury protocol. However, our data show that, although the mean number of neuromasts innervated by a single cell does not significant change after neurectomy, there is an increase in the number of neurons that co-innervate neighboring neuromasts after regeneration. The most promiscuous afferent cells were the younger neurons, and we found that these cells have the potential to innervate two or more neuromasts of the primary and secondary lateral lines at the same time, neuromasts whose hair cells are polarized with anteroposterior or dorsoventral orientation, respectively [69]. This finding is interesting, given it has been shown that afferent neurons contact target cells of identical polarity in adjacent primary neuromasts [70,71]. However, our results complement those of Sarrazin *et al.*[40], who showed that ablation of the pLL ganglion at 24 hpf and loss of all primary neurons was nonetheless followed by formation of a new ganglion (derived from a later developing secondary placode) whose neurons were able to coinnervate primary and secondary neuromasts. Therefore, we assume that the class of axons that are able to coinnervate primary and secondary neuromasts after axon regeneration in our work are likely from neurons originating from the secondary placode/ganglion; these cells have more recently been named follower neurons by Sato *et al.*[43].

We caution that, despite our findings, it is clear that the innervation process both during normal development and during regeneration is highly dynamic. Due to the difficulty of accurately following axons from 5 dpf onwards, we cannot rule out the possibility that the system may be further refined and some connections eventually become destabilized. Second, with respect to co-innervation of primary and secondary neuromasts by single axons after regeneration, we do not know whether those same neurons would have shown this behavior in the absence of regeneration. More experiments will be required to investigate this issue.

The lateralis system shows robust developmental somatotopy [26,44,72], though it is still unknown how this specificity is achieved. It has been shown that the information that is represented in this sensory map reflects the anteroposterior position of the neuromasts on the body surface [26]. Whether this organization is maintained after regeneration remains as an open question. Here, we showed that even if target organs are not reacquired with absolute fidelity, the anteroposterior innervation pattern of neuromasts by afferent neurons does not significantly change after regeneration of pLL nerve axons (Figure 2C). As we do not know the fate of the central connections, however, only behavioral studies will be able to determine if there is a functional restoration of this sensory system.

It has been shown that leader and follower neurons differ in their velocity of axonal extension during development [43]. However, it was difficult to discriminate between axons of these two types of cells. Using single-cell labeled fish, we provide evidence that some axons regrow significantly behind the pioneering axon supporting the idea of a pioneer-follower effect during regeneration, recapitulating this developmental feature of pLL nerve growth. Although the molecular basis of this behavior is unknown, axon competition for targets [73-76] and differential expression of guidance receptors and ligands of afferent neurons [46,57,77,78] have been proposed as candidate mechanisms to modulate the retinotopic organization in the visual system as well as synapse refinement at the neuromuscular junction. In the pLL nerve, how a regenerating axon is established as a leader or how isotypic pioneering-follower interactions contribute to rewire the system, require further study.

In fish, birds, and mammals, peripheral nerves are not only composed of afferent sensory axons but also include efferent pathways controlling diverse aspects of peripheral organ function. In this work, we did not focus on the relationship between afferent and efferent axons during peripheral nerve regeneration, as it has been shown that sensory efferent projections rely for growth on their association with pre-formed sensory afferent trajectories [79]. These authors also showed that this relationship between both types of axons is preserved from fish to mammals.

Intrinsic mechanisms have been suggested to promote nerve regeneration, but extrinsic local signals from immune and Schwann cells can also contribute to the process after peripheral nerve injury [14,22,65,80-82]. In particular, denervated Schwann cells can respond by dedifferentiation, inducing the expression of genes typical of an undifferentiated state, as well as neurotrophic factors that contribute to axon guidance [12,14,83]. Upon successful axonal regeneration, Schwann cells regain contact with axons and begin to differentiate once again into myelinating cells [10,84,85]. The pLL is a particularly attractive system to study axon-Schwann cell interactions, as it comprises one of the longest axon bundles in the zebrafish larva (and adult), and is wrapped by glial cells over its entire extension. Using larvae, we showed that the nerve regenerates completely 48 hpn growing along the chain of Schwann cells that lined the original nerve pathway. We had previously suggested that the remaining glia may provide a substrate for re-extension of the nerve [30]. Here, we provide additional evidence for this instructive and necessary role with a series of experiments. First, we performed neurectomy in *sdf1a* mutants in which the nerve has followed an erroneous path during development and Schwann cells are mislocalized. In this case, the nerve regenerates following the ectopic chain of Schwann cells and not the myoseptum. However, in this mutant, interneuromastic cells are also present in the aberrant pathway and could participate in guidance of the regenerating axons, as has been shown [86]. We thus searched for a scenario where larvae had normal primordium migration and, in consequence, interneuromastic cells along the myospetum, but absence of Schwann cells. By exposing fish to AG1478 at different time points, we could partially disrupt Schwann cell migration and could therefore generate pLL nerves covered in part with Schwann cells leaving others devoid of these cells. In the absence of glial cells, neurites arising from severed axons were able to grow, but they did so erratically and most often failed to reach their sensory cell targets. In a recent publication [31], it has been shown in the laser ablated pLL nerve of adult fish that ablation of a subset of Schwann cells located in the somite adjacent to the cut only provokes a delay in the onset of reinnervation of neuromasts. In this context, the nerve regrows very tortuously through the gap lacking Schwann cells, but later regains contact with distally located glial cells and returns to a normal coherent pathway eventually reaching the tip of the tail. These, and our own observations, show that pLL axons can regrow in isolation, but need the guidance and support of Schwann cells in order to properly navigate their original path and reinnervate their targets.

Immediately after neurectomy in the pLL nerve, there is a period of latency in which no axonal growth occurs [30,31], this work. We found that the inhibition of ErbB signaling in neurectomized larvae extends the period of latency without affecting the velocity of axonal growth after it has begun. This treatment also induces the defasciculation of neurectomized and non-neurectomized pLL axons. It has been recently shown that the inhibition of ErbB signaling pathway in larvae using 5 μM AG1478 does not kill Schwann cells [87]. However, it is known that ErbB signaling is required for Schwann cell process extension [88]. We suggest that the delay in regenerative axonogenesis induced by AG1478 treatment occurs because Schwann cells are less motile and cannot efficiently interact with the regrowing axons. In turn, this prevents the growing axons from rapidly crossing the gap created by the neurectomy procedure. We conclude that the onset of regeneration depends of the interaction between regrowing axons and denervated Schwann cells, whereas the rate of regeneration once growth of the neurites starts, does not depend on this interaction.

An aspect that has not been addressed satisfactorily in pLL nerve regeneration is the role of the immune system. We showed previously that there is a strong inflammatory response after neurectomy, both using laser ablation [30] and electroablation [52]. In the absence of leukocytes, axonal debris removal is delayed and regeneration of a neurectomized nerve takes longer to complete. In contrast, removal of Schwann cells did not affect debris removal, indicating that Schwann cells, immune cells, and possibly other cell types can all contribute to fragment clearance and establishing a proper environment for axon regeneration after damage [30]. It will be important to dissect the relative contributions of all implicated cell types in the process of axon regeneration in this model.

In our model, Schwann cells did not die but they respond to neurectomy by losing expression of MBP. Using cell transplantation experiments, we were also able to observe engulfment of axonal debris by Schwann cells in an *in vivo* context, as was previously suggested [17]. Surprisingly, Schwann cells remain vacuolated at 24 hpn (Figure 7H). Recent findings have suggested that these glial cells supply exosomal vesicles to axons after axonal damage as well as during axonal regeneration, improving axonal regeneration in dorsal root ganglion neurons [89]. This could represent a general mode of action of the Schwann cells contributing to regeneration.

In zebrafish, from 3 to 4 dpf, Schwann cells are in the process of radial sorting to the promyelinating stage [88], reviewed in [90]. At this point the number of promyelinating cells are regulated by proliferation of immature cells instead of cell death [61,88,90] in order to match Schwann cells and axon numbers. In contrast, in the peripheral nervous system of mice, proliferation in the early postnatal period is followed by cell death before the onset of myelination [91]. Although we do not see a wave of Schwann cell proliferation in response to loss of axonal contacts in 5 dpf zebrafish larvae as occurs in mammals [58,92], we found that Schwann cells remain in a proliferative state in neurectomized larvae, whereas in controls they are differentiated and no longer proliferative. Our finding raises the question of whether fish differ from mammals in terms of the control of Schwann cell number or if the difference is more related to the developmental stage at which neurectomy is carried out. It will eventually be interesting to address whether proliferation of Schwann cells has a different impact on nerve regeneration in both models [91].

## Conclusion

We provide a description of the temporal events triggered by peripheral nerve injury in the pLL nerve of zebrafish larvae at the single cell level. We focused on the nerve-glia interaction and also on the axonal behavior of damaged neurons. We show that Schwann cells located just posterior to the gap have an important role in the onset of nerve regeneration and that these cells are critical for correct guidance of regenerating axons but not for growth itself. In addition, the organization of the pLL axons at 3 dpf is not precisely replicated after injury but is most likely able to restore a measure of somatotopy after reinnervation of mechanosensory cells. Our results invite an exploration of the molecular mechanisms involved in re-establishment of a functional sensory system in a vertebrate after peripheral nerve damage.

## Methods

### Zebrafish husbandry and transgenic lines

Zebrafish (*Danio rerio*) embryos were obtained by natural spawning of the following transgenic strains: *Tg(neuroD:EGFP)*^*nl1*^[54], *Tg(brn3c:GAP43-GFP)*^*s356t*^[93], *Tg(cxcr4b:mRFP)*^*ump1*^[94], *Tg(neurod:TagRFP)*^*w69*^ (kindly provided by Dr. David Raible), *Tg(foxd3:GFP)*^*zf104*^[28], *Tg(8.0cldnb:lynEGFP)*[93], *Tg (Ubi:zebrabow:cherry)*[95], and *sdf1α*^*u766*^ mutants (Mardones *et al.*, in preparation). The embryos were staged according to Kimmel *et al.*[96]. We express larval ages in hpf or dpf.

Fertilized eggs were raised in petri dishes containing E3 medium (5 mM NaCl, 0.17 mM KCl, 0.33 mM CaCl2, 0.3 mM MgSO4, and 0.1% methylene blue) until 24 hpf. From this stage to 5 dpf the embryos were incubated in E3 medium supplemented with 0.003% of phenylthiourea (PTU) to prevent pigmentation. After 5 dpf, the larvae were incubated in E3 medium lacking PTU to avoid excessive toxicity. Most of the experiments were carried out in 72 hpf larvae, because at this stage the primary lateral line is completely developed and functional [53]. All procedures complied with national guidelines of the Animal Use Ethics Committee of the University of Chile and the Bioethics Advisory Committee of Fondecyt-Conicyt (the funding agency for this work).

### DNA injection and neurectomy

Approximately 5 nl of a solution of *HuC:mem-TdTomato* (40 ng/μL) DNA (obtained from Dr. Hernán López-Schier) or 5 nl of a solution of *pE46:GFP* (20 ng/μL) DNA were injected into one-cell-stage *Tg(neuroD:EGFP)* or *Tg(neurod:TagRFP)* embryos, respectively. The *pE46:GFP* DNA was generated using a putative enhancer of the mouse *delta like 1* gene (Valdivia and Allende, unpublished; construct available upon request). Cloning of the enhancer of the *delta like 1* gene was carried out by PCR with the forward GGGCGCCTCTGACCTCCCACA and reverse CTCCGGGGCCAGGTGGGGCT primers using mouse genomic DNA as template. Because of variegated DNA integration and expression, this approach allowed us to label single lateral line neurons in injected larvae.

Injected larvae were neurectomized at 3 dpf as described by Moya-Díaz *et al.*[52]. Briefly, 3 or 5 dpf larvae were anesthetized with 0.01% tricaine and mounted in rectangular plates sealed with low melting point agarose (0.75%) dissolved in E3 medium. Once the agarose was set, the embryos were neurectomized using a tungsten electrode connected to a power source under the following conditions: 1 pulse of 1.5 s duration and 17 μA of current intensity. After this procedure the larvae were dismounted and analyzed under a fluorescent scope. Successfully neurectomized larvae were selected and maintained at 28°C. We analyzed the behavior of the axons during regeneration at different time points defined as hpn.

### Conditional inhibition of ErbB signaling

AG1478 is a competitive kinase inhibitor which is routinely used in zebrafish to block the ErbB signaling pathway [30,61,97,98]. A final concentration of 5 μM AG1478 (Calbiochem) in 0.05% DMSO was added to larvae in E3 medium from ages 10 hpf to 58 hpf in order to block ErbB signaling and thus Schwann cell migration. A final concentration of 3.5 μM of AG1478 in 0.035% DMSO added to embryos between 24 hpf and 58 hpf resulted in a partial inhibition of Schwann cells migration. A 5 μM concentration of AG1478 in 0.05% DMSO was used in 60 hpf to 5 dpf larvae to block ErbB signaling 12 h before neurectomy and during the regeneration process of the pLL nerve.

### Transplantation experiments

Donor and host embryos were raised in E3 medium until the high stage (3 1/3 h). At this stage both groups of embryos were dechorionated by incubation with pronase (0.2 mg/mL) and maintained in Holtfreter’s solution (NaCl 59 mM; KCl 0,67 mM; CaCl2 0,9 mM; MgSO4 0,81 mM; NaHCO3 2,38 mM) with penicillin/streptomycin (5000 U/L; 100 mg/L; Sigma). Approximately 10 to 20 donor cells were aspirated and transplanted into host embryos at the same stage using a micropipette connected to a 1 mL syringe as described previously [99]. Host embryos were screened for the presence of donor cells in the pLL at 48 hpf.

### BrdU labeling and inmunohistochemistry

For BrdU incorporation, control *Tg(foxD3:GFP)* larvae were maintained in a solution of 10 mM BrdU and 5% DMSO dissolved in E3 medium at 28°C for periods of 3 h starting at different time points: 72 hpf, 84 hpf, 90 hpf, 4 dpf, 6 dpf, and 7 dpf after Brdu incorporation larvae were fixed in 4% PFA and dehydrated in methanol until processed for immunostaining. To analyze the proliferative response of Schwann cells to denervation, we incubated control and 5 dpf neurectomized *Tg(foxD3:GFP)* larvae in a solution of 10 mM BrdU and 5% DMSO dissolved in E3 medium at 28°C for periods of 3 h starting at different time points: 6 dpf (24 hpn) and 7 dpf (48 hpn). In these experiments, we employed eight to 10 larvae per condition and repeated this experiment three times in order to obtain approximately 30 individuals per time point in each group.

Whole-mount immunohistochemistry was performed using rabbit anti-GFP (1:500 Invitrogen, A11122), mouse anti-GFP (1:500; Millipore, MAB3580), mouse anti BrdU (1:500; Roche, 11170376001), rabbit anti-MBP (1:50; kindly provided by Dr. William Talbot), Alexa 488 goat anti-mouse (1:2,000; Invitrogen, 11029), Alexa 594 goat anti-rabbit (1:2,000; Invitrogen, A31632), Alexa 488 goat anti-rabbit (1:2,000; Invitrogen, 11034), Alexa 594 goat anti-mouse (1:2,000; Invitrogen, A11032) following standard procedures. Briefly, larvae were rehydrated from methanol, rinsed in 0.1% PBS-Tween, permeabilized with proteinase K (40 μg/mL, 40 min for 3 dpf larvae and 70 min for 5 to 7 dpf larvae), washed and refixed in 4% PFA, transferred to blocking solution (2% normal goat serum, 1% DMSO, 1 mg/mL BSA in PBS 0.1%Tween) for 45 min. Larvae were then incubated overnight at 4°C with primary antibodies, washed with PBS 0.1% Tween and incubated 2 h at room temperature with secondary antibodies dissolved in blocking solution. For MBP immunohistochemistry the larvae were treated with three cycles of cold sodium citrate buffer (10 mM, pH 6) of 10 min each, followed by 1 min with citrate at 90°C after proteinase K treatment.

### Image acquisition and time-lapse imaging

pLL axons were imaged in stable transgenic *tg(neuroD:GFP)* or *tg(neuroD:tagRFP)* zebrafish larvae and in transient expressing *HuC:mem-TdTomato* or *pE46:GFP* larvae. Embryos were anaesthetized in 0.01% tricaine and mounted in a sealed agarose plates. A Zeiss confocal microscope (Zeiss LSM 510 meta) was used to image lateral line nerve and Schwann cells, with 10× or 20× objectives. The assembly of the images to display the full larvae was done by using Adobe Photoshop CS5.

For time-lapse analysis, embryos were imaged at various intervals after axotomy for about 12 h on a confocal microscope with a 10× or 20× dry objective (Zeiss LSM 510 meta). Approximately 15 confocal sections of recommended thickness according to the objective were gathered at each time point into maximum projections and compiled into movies with ImageJ software. Embryos were maintained at 28°C with a heat stage throughout imaging.

### Quantifications and statistical analysis

ImageJ was used to quantify the winding index of the nerve (calculated as the relationship between trajectory vs maximal distance) under three different conditions (control, partial, or total absence of Schwann cells). The maximal distance of the nerve was defined as the theoretical length between the pLL ganglion and axonal growth cones of the nerve following the normal pathway through the mioseptum. Trajectory means the real length between the pLL ganglion and axonal growth cones of the nerve following exactly the same pathway of the regrowing nerve.

The *cell counter* plugin of ImageJ was used to quantify the number of double labeled *foxD3:GFP*^*+*^*/*BrdU^+^ cells in control and neurectomized larvae at different time points. We used ANOVA for treatment comparison or an equivalent nonparametric method (Kruskal-Wallis), depending on the structure of the data. Additionally we used a two-way ANOVA when the measured parameter depended on two factors. The significance level was *P* <0.05 for all treatments. All data analysis was performed using Prism 5.0b (GraphPad Prism Software, Inc., USA).

## Abbreviations

dpf: Days post fertilization; dpn: Days post neurectomy; GFP: Green fluorescent protein; hpf: Hours post fertilization; hpn: Hours post neurectomy; MBP: Myelin basic protein; pLL: Posterior lateral line; RFP: Red fluorescent protein.

## Competing interests

The authors declare that they have no competing interests.

## Authors’ contributions

MLC designed the experiments, carried out the nerve regeneration studies, performed the characterization of Schwann cell behavior, and drafted a first version of the manuscript. CMK carried out the nerve regeneration studies in *sdf1* mutant larvae. MS carried out cell transplantation experiments. LV generated the *pE46:GFP* construct, isolated the *sdf1* mutant line, and helped to draft the first version of the manuscript. MA conceived of the study, participated in its design, and prepared together with MLC the final draft of the manuscript. All authors read and approved the final manuscript.

## Supplementary Material

Additional file 1 **Visualization of single neurons through time.** The pLL afferent neurons labeled by integration of HuC:memdTomato or *pE46:GFP* DNA were imaged 1 h before neurectomy (1 hbn) and after nerve regeneration (3, 24, 48 hpn). Two different examples are shown: (A-D) and (A’-C’). D’ corresponds to the inset in C’. The peripheral projection of every single pLL neuron follows a particular and unique path into the pLL nerve that makes it easily distinguishable from others (arrowheads). At 3 to 5 hpn (B) the distal portion of the pLL nerve has undergone degeneration (inset in B shows the remains of the severed axon in the posterior trunk) whereas the proximal stump remains intact. At, 3, 24, or 48 hpn, the neuron can be easily recognized as the labeled cell shows the exact same pathway as before neurectomy (arrowheads). Note that single neurons and their axons are more easily visualized at 3 dpf than at 5 dpf (compare A’ to C’, respectively). Click here for file

Additional file 2 **Changes in the innervation pattern of the pLL afferent neurons after neurectomy.** The specific innervation of neuromasts by single-labeled pLL ganglion neurons was recorded before neurectomy and after nerve regeneration (48 hpn); each neuron belongs to a different larva as was previously described (see Figure 2). The chart on the left shows the percentage of neurons that innervate a single (purple) vs. multiple neuromasts (green) before neurectomy. In the central graph, the green and purple outlines indicate the distribution of innervation patterns before neurectomy, whereas the fill color indicates the behavior of afferent neurons after 48 hpn. The decision to innervate one or more target organs was independent of the previous situation of the neuron. The graph on the right shows the final distribution of cells that innervate a single (purple) vs. multiple neuromasts (green) 48 h post neurectomy. Click here for file

Additional file 3 **Axonal and Schwann cell behavior during repair of the neurectomy lesion site.** Double transgenic larvae (Schwann cells in green, axons in red: see Methods) were neurectomized at 72 hpf and were imaged for 12 h using time-lapse fluorescence microscopy. Top movie shows merged channels while central and bottom show red and green channels, respectively. Anterior (where the pLL ganglion is located, that is, proximal) is to the left. Note that Schwann cells distal to the lesion site are more motile and extend processes towards the growing axons before a connection between both sides is established. Also note that the axons (and not Schwann cells) pioneer the crossing of the gap made by electroablation from the proximal side. Click here for file

Additional file 4 **MBP expression recovery in larvae neurectomized at 3 dpf. (A) ***tg(foxd3:GFP)* larvae were neurectomized at 3 dpf. After 48 hpn, the larvae were fixed and processed for anti-MBP labeling. At this time, MBP expression reappears in a proximal to distal wave. B: At 5 dpn, MBP expression is detected through the entire pLL nerve. Scale: A-C: 100 μm. Click here for file

Additional file 5 **Loss of Schwann cell differentiation markers after denervation at 5 dpf.** Five-day-old *tg(foxD3:GFP)/tg(NeuroD:RFP)* double transgenic larvae were left untreated or were neurectomized and observed 2 dpn. In these fish, Schwann cells are labeled by green and the nerve by red fluorescence. In neurectomized fish, at 48 hpn, the regrowing nerve almost reaches the tip of the tail (arrowhead in **(A)**, inset). At the same time, a distal decrease in GFP expression is observed **(B)** compared to age-matched non-neurectomized controls **(C)** (compare insets that show enlarged image of trunk and tail). The same experiment carried out with 3-day-old fish showed a similar result. The dotted square in **(D)** shows the area of the fish imaged in **(E, F)**. E shows Schwann cells in a larva 24 hpn; F shows the same area in a control larva. Scale: E, F: 200 μm; A-C, inset in B, inset in C: 100 μm. Click here for file

Additional file 6 **Aberrant pLL axon regeneration in the complete absence of Schwann cells.** neuroD:EGFP larvae were treated with AG1478 from 10 to 58 hpf, were neurectomized, and the regenerating pLL nerve was imaged under epifluorescence microscopy from 29 hpn to 34 hpn The pioneering axon shows aberrant behavior as it explores areas outside of the horizontal myoseptum and fails to advance posteriorly; the nerve becomes progressively defasciculated. Click here for file

Additional file 7 **Impairment of axonal regeneration in 3 dpf larvae treated with AG1478 from 10 hpf to 58 hpf.** A double transgenic neuroD:EGFP; Brn3c:GAP43-GFP larva was treated with AG1478 from 10 to 58 hpf and was neurectomized at 72 hpf. The behavior of regrowing pLL axons was imaged under confocal microscopy from 29 hpn to 34 hpn. The movie shows the growth behavior of an axon that differs from that observed in Additional file 4; in this case, the axon extends a few micras but then retracts, remaining in the same position for long periods of time (see Figure 9A-D). Click here for file

Additional file 8 **Impairment of axonal regeneration in 3 dpf larvae treated with AG1478 from 24 to 58 hpf.** A triple transgenic neuroD:EGFP/foxD3:GFP/Cxcr4:mCherry larva was treated with AG1478 from 24 to 58 hpf in order to obtain a partial absence of Schwann cells. The larva was neurectomized and imaged from 26 to 35 hpn. The pLL nerve and Schwann cells are pseudocolored in yellow, whereas the neuromasts and interneuromastic cells located between them are labeled in red. At the beginning of the sequence, there are two glial cells located distal to the regrowing axons. During regeneration, these isolated Schwann cells are contacted by the axons which continue to grow along the myoseptum following the trail of interneuromastic cells (see Figure 9A’-D’). Click here for file

Additional file 9 **Schwann cells migrate with regenerating axons. (A, B)** In non-axotomized larvae treated with 3.5 μM AG1478 from 24 to 58 hpf, Schwann cell migration is arrested and these cells remain immotile and confined to the horizontal myoseptum. **(C, D)** In neurectomized fish that have been treated with AG1478 as above, Schwann cells are often found at ectopic positions as they follow the erratic path of the regrowing nerve. Scale: A-D: 200 μm. Click here for file
