# Supplementary figures and images for: Axon-Schwann cell interactions during peripheral nerve regeneration in zebrafish larvae
Source: Neural Dev. 2014 Oct 17;9:22. doi: 10.1186/1749-8104-9-22 (PMC4214607; doi:10.1186/1749-8104-9-22)

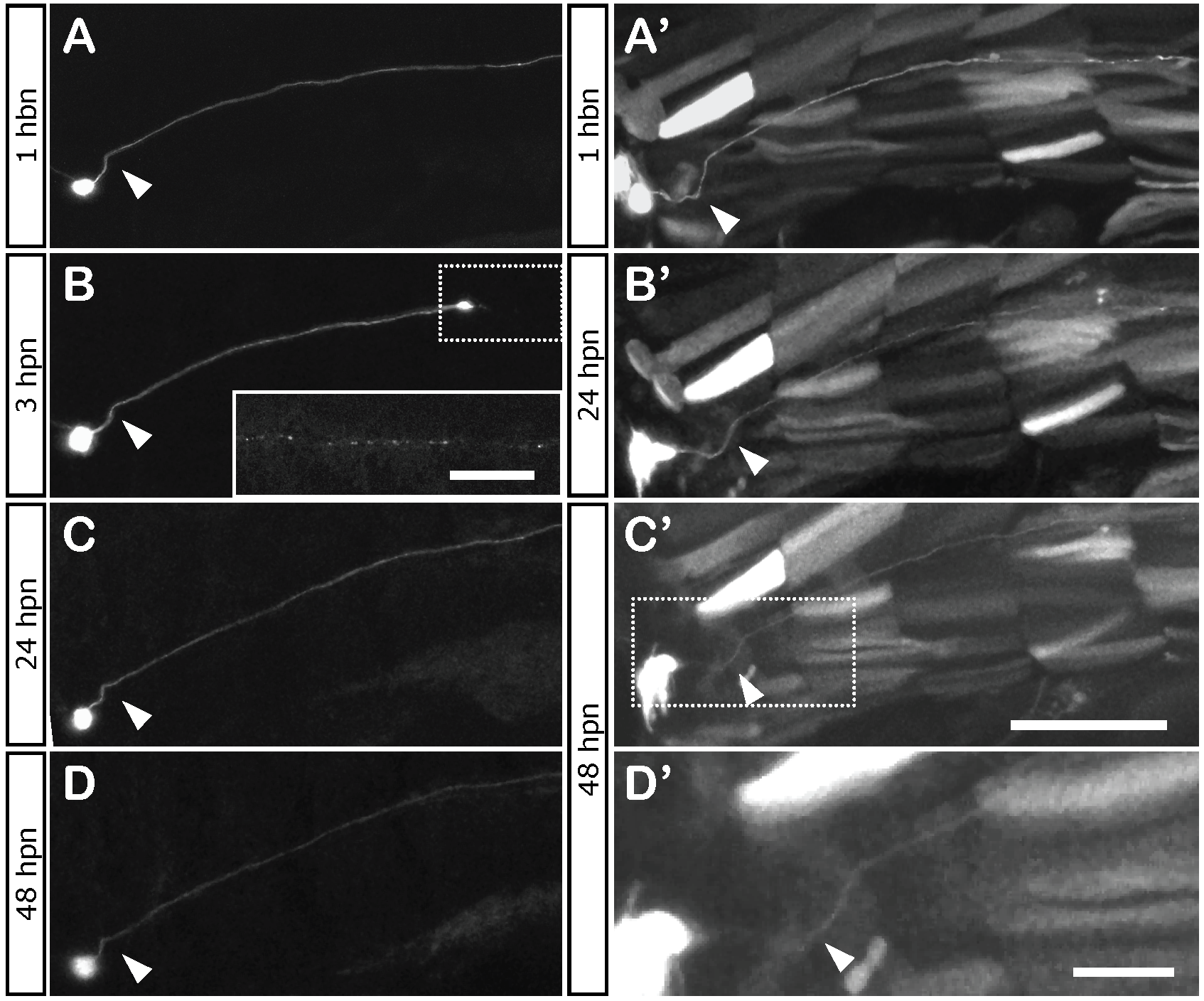

Supplement: Additional file 1 — Visualization of single neurons through time. The pLL afferent neurons labeled by integration of HuC:memdTomato or pE46:GFP DNA were imaged 1 h before neurectomy (1 hbn) and after nerve regeneration (3, 24, 48 hpn). Two different examples are shown: (A-D) and (A’-C’). D’ corresponds to the inset in C’. The peripheral projection of every single pLL neuron follows a particular and unique path into the pLL nerve that makes it easily distinguishable from others (arrowheads). At 3 to 5 hpn (B) the distal portion of the pLL nerve has undergone degeneration (inset in B shows the remains of the severed axon in the posterior trunk) whereas the proximal stump remains intact. At, 3, 24, or 48 hpn, the neuron can be easily recognized as the labeled cell shows the exact same pathway as before neurectomy (arrowheads). Note that single neurons and their axons are more easily visualized at 3 dpf than at 5 dpf (compare A’ to C’, respectively). [file 1749-8104-9-22-S1.tiff]

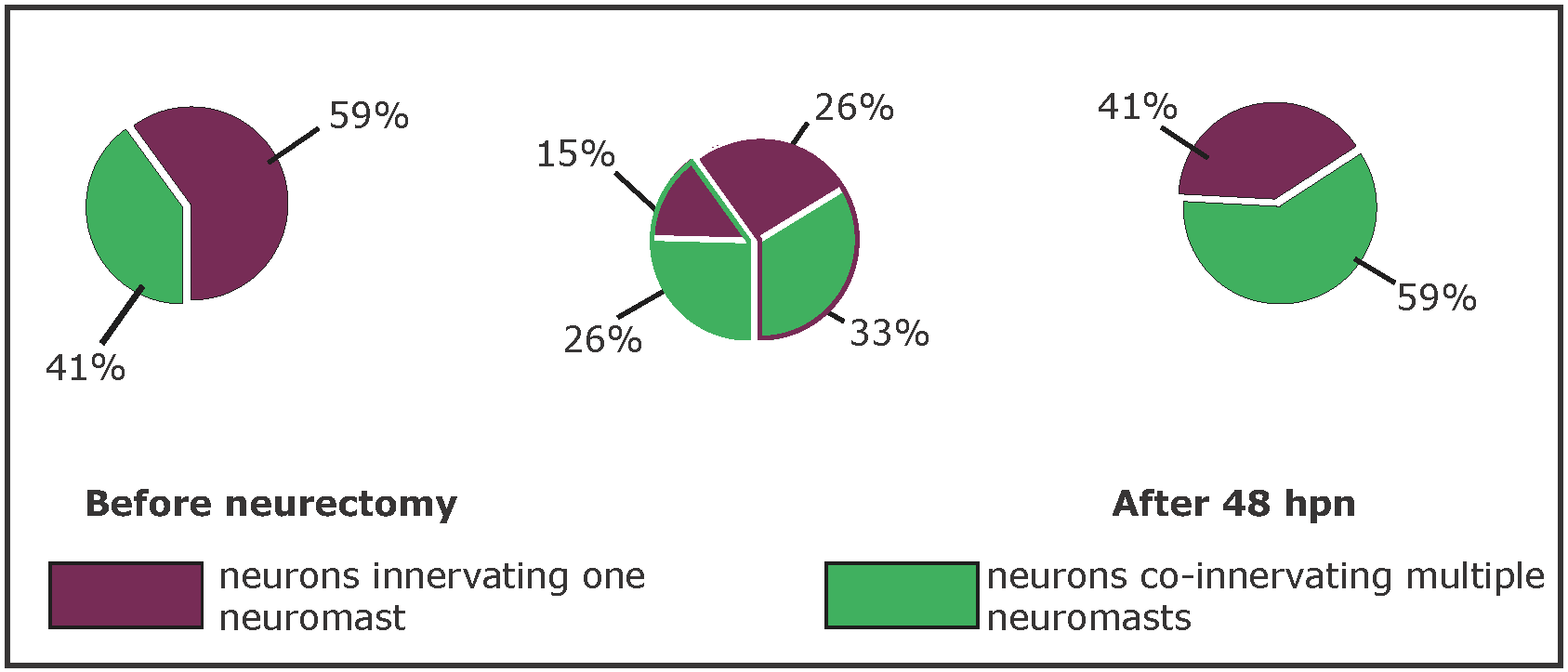

Supplement: Additional file 2 — Changes in the innervation pattern of the pLL afferent neurons after neurectomy. The specific innervation of neuromasts by single-labeled pLL ganglion neurons was recorded before neurectomy and after nerve regeneration (48 hpn); each neuron belongs to a different larva as was previously described (see Figure 2). The chart on the left shows the percentage of neurons that innervate a single (purple) vs. multiple neuromasts (green) before neurectomy. In the central graph, the green and purple outlines indicate the distribution of innervation patterns before neurectomy, whereas the fill color indicates the behavior of afferent neurons after 48 hpn. The decision to innervate one or more target organs was independent of the previous situation of the neuron. The graph on the right shows the final distribution of cells that innervate a single (purple) vs. multiple neuromasts (green) 48 h post neurectomy. [file 1749-8104-9-22-S2.tiff]

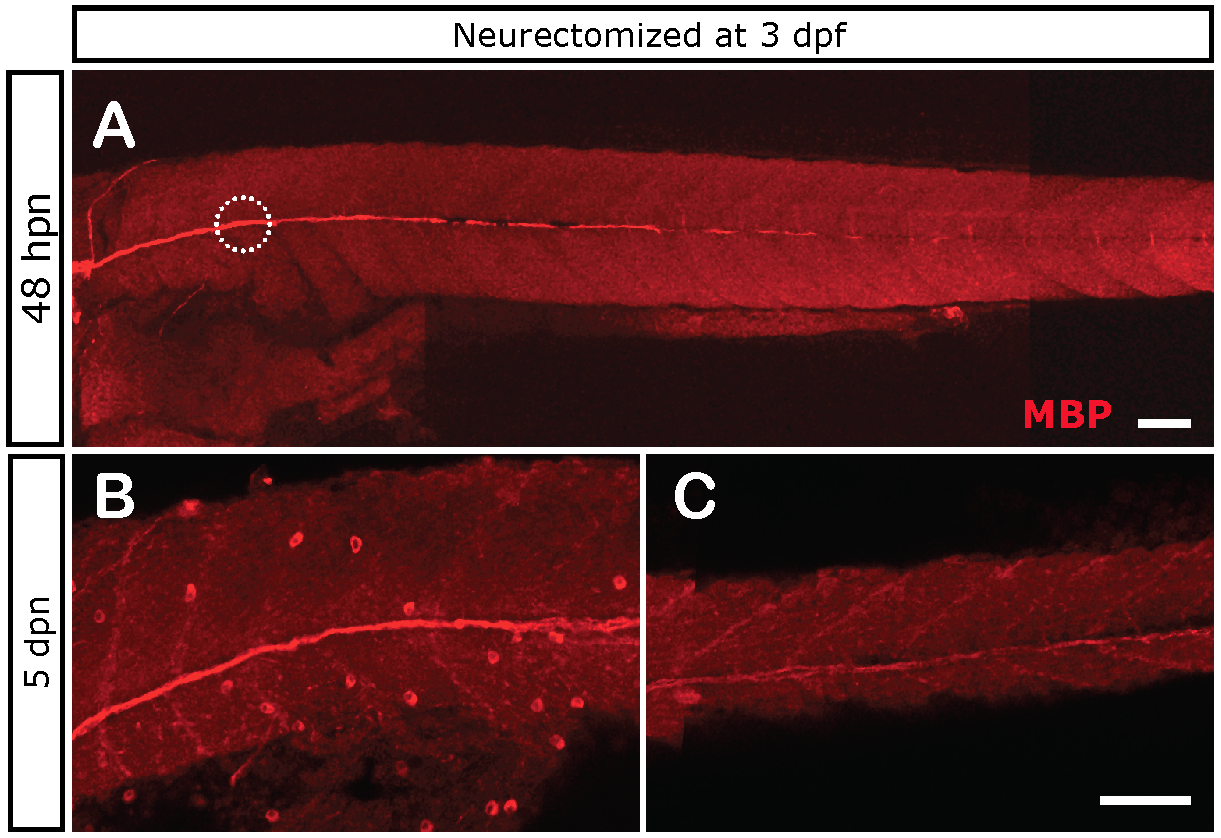

Supplement: Additional file 4 — MBP expression recovery in larvae neurectomized at 3 dpf. (A) tg(foxd3:GFP) larvae were neurectomized at 3 dpf. After 48 hpn, the larvae were fixed and processed for anti-MBP labeling. At this time, MBP expression reappears in a proximal to distal wave. B: At 5 dpn, MBP expression is detected through the entire pLL nerve. Scale: A-C: 100 μm. [file 1749-8104-9-22-S4.tiff]

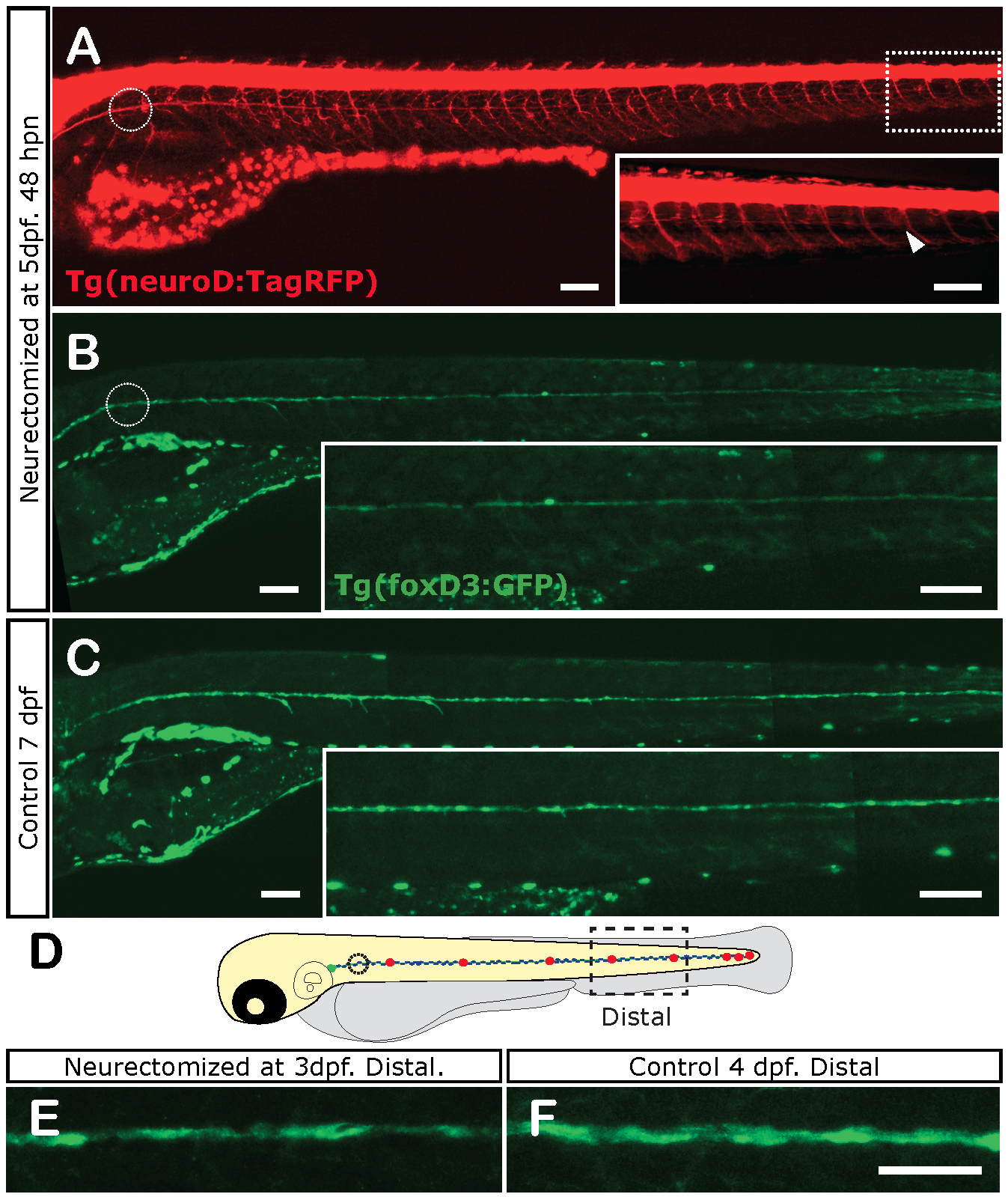

Supplement: Additional file 5 — Loss of Schwann cell differentiation markers after denervation at 5 dpf. Five-day-old tg(foxD3:GFP)/tg(NeuroD:RFP) double transgenic larvae were left untreated or were neurectomized and observed 2 dpn. In these fish, Schwann cells are labeled by green and the nerve by red fluorescence. In neurectomized fish, at 48 hpn, the regrowing nerve almost reaches the tip of the tail (arrowhead in (A), inset). At the same time, a distal decrease in GFP expression is observed (B) compared to age-matched non-neurectomized controls (C) (compare insets that show enlarged image of trunk and tail). The same experiment carried out with 3-day-old fish showed a similar result. The dotted square in (D) shows the area of the fish imaged in (E, F). E shows Schwann cells in a larva 24 hpn; F shows the same area in a control larva. Scale: E, F: 200 μm; A-C, inset in B, inset in C: 100 μm. [file 1749-8104-9-22-S5.tiff]

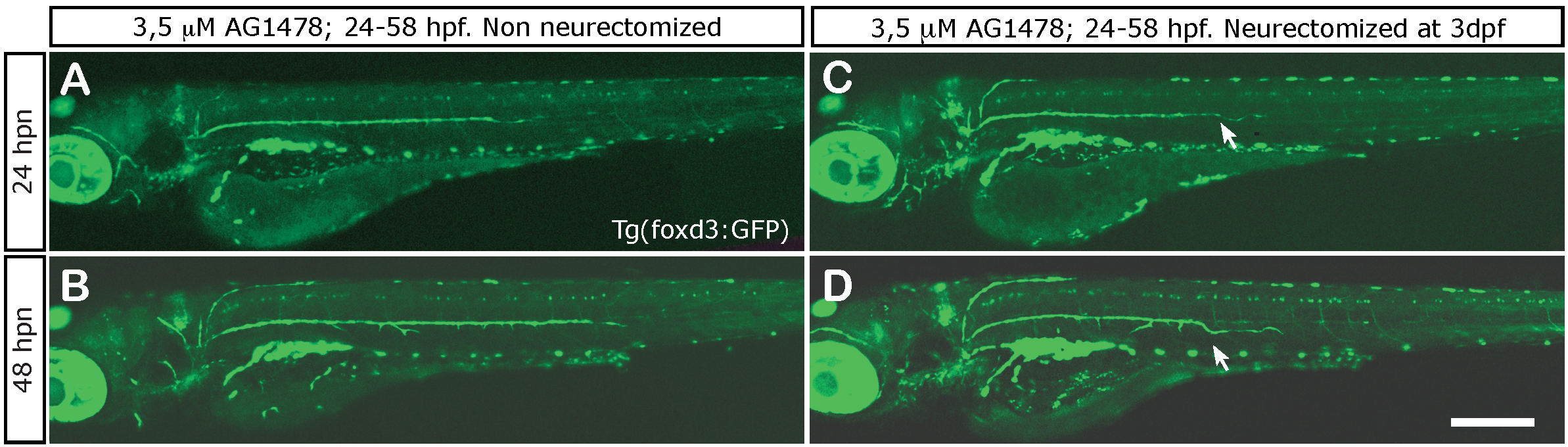

Supplement: Additional file 9 — Schwann cells migrate with regenerating axons. (A, B) In non-axotomized larvae treated with 3.5 μM AG1478 from 24 to 58 hpf, Schwann cell migration is arrested and these cells remain immotile and confined to the horizontal myoseptum. (C, D) In neurectomized fish that have been treated with AG1478 as above, Schwann cells are often found at ectopic positions as they follow the erratic path of the regrowing nerve. Scale: A-D: 200 μm. [file 1749-8104-9-22-S9.tiff]
